# A narrow T cell receptor repertoire instructs thymic differentiation of MHC class Ib–restricted CD8^+^ regulatory T cells

**DOI:** 10.1172/JCI170512

**Published:** 2024-01-02

**Authors:** Hye-Jung Kim, Hidetoshi Nakagawa, John Y. Choi, Xuchun Che, Andrew Divris, Qingshi Liu, Andrew E. Wight, Hengcheng Zhang, Anis Saad, Zhabiz Solhjou, Christa Deban, Jamil R. Azzi, Harvey Cantor

**Affiliations:** 1 Department of Cancer Immunology & Virology, Dana Farber Cancer Institute, Boston, Massachusetts, USA.; 2 Department of Immunology and; 3 Department of Medicine, Harvard Medical School, Boston, Massachusetts, USA.; 4 Transplant Research Center, Brigham and Women’s Hospital, Boston, Massachusetts, USA.; 5 Department of Immunology, School of Basic Medical Sciences, Tianjin Medical University, Tianjin, China; 6 Department of Immunology, School of Basic Medical Sciences, Zhengzhou University, Zhengzhou, China.

**Keywords:** Immunology, T cell receptor

## Abstract

Although most CD8^+^ T cells are equipped to kill infected or transformed cells, a subset may regulate immune responses and preserve self-tolerance. Here, we describe a CD8 lineage that is instructed to differentiate into CD8 T regulatory cells (Tregs) by a surprisingly restricted set of T cell receptors (TCRs) that recognize MHC-E (mouse Qa-1) and several dominant self-peptides. Recognition and elimination of pathogenic target cells that express these Qa-1–self-peptide complexes selectively inhibits pathogenic antibody responses without generalized immune suppression. Immunization with synthetic agonist peptides that mobilize CD8 Tregs in vivo efficiently inhibit antigraft antibody responses and markedly prolong heart and kidney organ graft survival. Definition of TCR-dependent differentiation and target recognition by this lineage of CD8 Tregs may open the way to new therapeutic approaches to inhibit pathogenic antibody responses.

## Abstract 2

CD8+ T regs express a restricted T cell receptor repertoire, instructing developing thymocytes to differentiate into regulatory CD8+ T cells.

## Graphical abstract

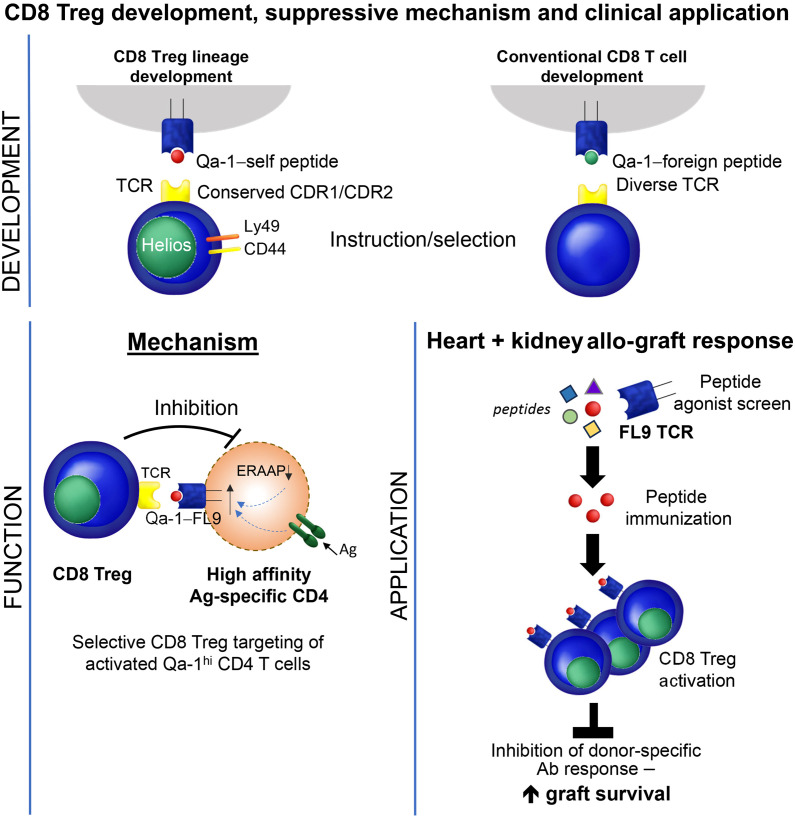

## Introduction

The immune system has evolved complex mechanisms that allow efficient destruction of microbial pathogens while sparing the host’s own tissues. Maintenance of this balance depends, in part, on regulatory T cells (1). Although most CD8^+^ T cells are equipped to kill cells infected by microbial invaders, there is increasing evidence that some CD8^+^ T cells are genetically programmed to suppress immune responses (reviewed in [2–4]). A subset of murine and human CD8 T cells that mediate regulatory activity is invested in approximately 5% of CD8 T cells that express a characteristic triad of surface receptors (CD44, CD122 and Ly49/KIR) (5–7) that carry out perforin-dependent killing of chronically activated and autoreactive CD4 cells (6, 8). Analysis of autoimmune disorders has revealed that CD8 T regulatory cells (CD8 Tregs) inhibit pathogenic responses through recognition of self-peptides associated with MHC class Ia or class Ib (MHC-E: mouse Qa-1 and human HLA-E) (2, 6) expressed by target CD4^+^ T helper cells. Here we define class Ib–restricted CD8 Tregs according to T cell receptor (TCR) expression, thymus-dependent development, and specific recognition mechanisms that eliminate activated CD4 T cells that upregulate Qa-1-self–peptide complexes.

Cell surface expression of Qa-1–peptide complexes by activated T cells depends on trimming by several enzymes, including an endoplasmic reticulum aminopeptidase associated with antigen processing (ERAAP), which digests larger peptides into 9-or-10mers that efficiently bind to Qa-1. Diminished or defective ERAAP activity associated with chronic or robustly activated CD4 T cells is marked by increased expression of a Qa-1–associated self-peptide, termed FL9 (9, 10). Chronically activated CD4 T cells also express self-peptides derived from the Hsp60 protein that associate with Qa-1 and activate CD8 Tregs (11). To define the TCRs used for recognition of these pQa-1 complexes, we cloned and analyzed 2 sets of TCRs expressed by CD8 Tregs that recognize the 2 structurally distinct self-peptides — FL9 and Hsp60_p216_ — complexed to Qa-1 (10, 11). This analysis revealed enrichment of TRAV and TRBV genes encoding highly conserved CDR1 and CDR2 regions and a highly variable CDR3 sequence that may contribute to specific recognition of either self-peptide. Analysis of TCR transgenic (Tg) CD8 T cells that expressed these receptors confirmed that MHC (Qa-1) recognition in the thymus may allow CD8 Tregs to escape negative selection by self-peptides and efficiently recognize and eliminate Qa-1^+^ CD4^+^ Th cells in peripheral tissues. Mutation or deletion of Qa-1 severely impaired intrathymic development and peripheral survival and suppressive function of CD8 Tregs.

Identification and expression of TCRs expressed by a regulatory lineage of Qa-1–restricted CD8 T cells also allowed definition of synthetic variants of the FL9 self-peptide that were used to efficiently activate and expand CD8 Tregs and suppress pathogenic CD4 cells during an immune response. We used this peptide-based approach to inhibit T follicular helper cell–driven (Tfh-driven) alloantibody responses and prolong survival of fully MHC-mismatched heart and kidney allografts in a preclinical model of heart and kidney transplantation. Insight into TCR usage, specificity, and function of CD8 Tregs may open the way to new therapeutic approaches to dampen pathogenic immune responses in inflammatory or autoimmune disorders.

## Results

### Identification of TCRs specific for Qa-1 self–peptide complexes.

Insight into the specialized function of both class Ia- and class Ib–restricted CD8 Tregs has relied mainly on isolation of both subsets of CD8 Tregs based on expression of a triad of shared surface markers — CD44, CD122, and Ly49. Here we distinguish class Ib (Qa-1–restricted) CD8 Tregs from class Ia–restricted Tregs according to expression of TCR specific for 2 structurally unrelated self-peptides — FL9 and Hsp60 — that are presented by Qa-1 and allow specific targeting of CD4 cells by CD8 Tregs (2, 10, 11).

**Figure 1:**
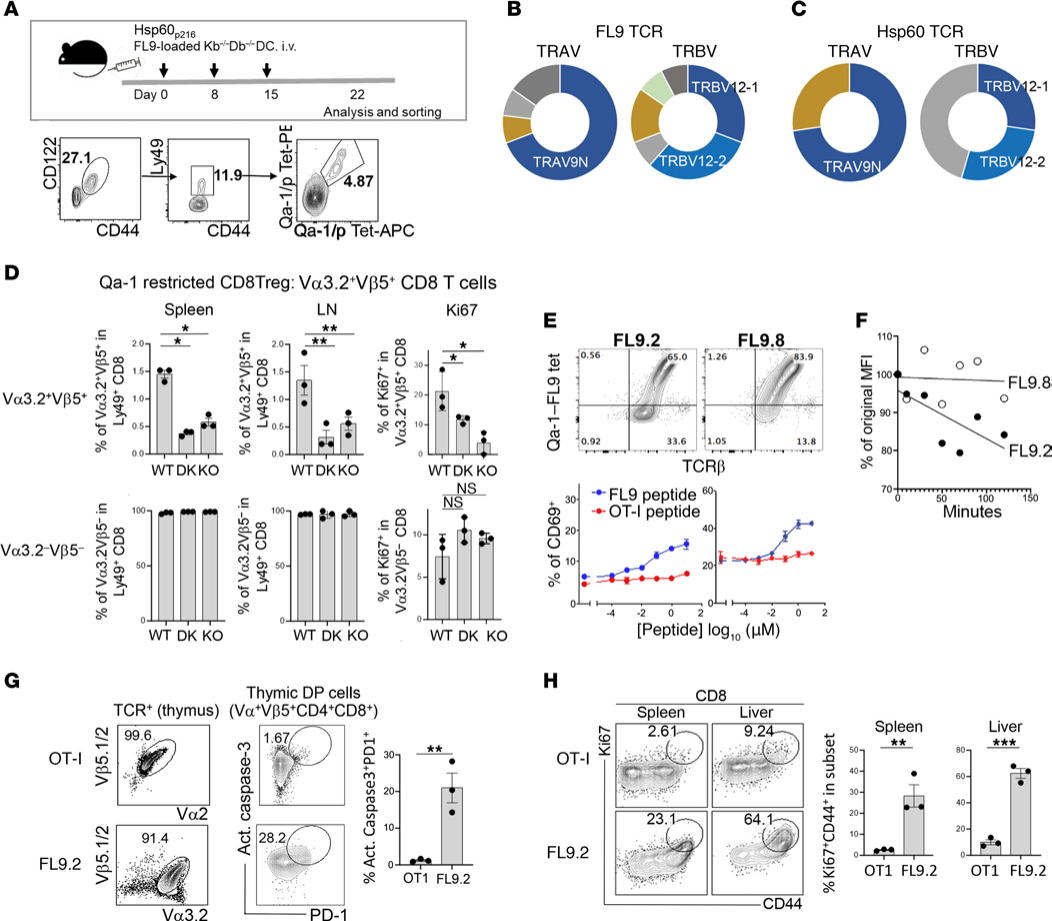
Identification of Qa-1–FL9–specific TCR. (**A**) WT B6 mice were immunized with Kb^–/–^Db^–/–^ DC loaded with FL9 peptide on days 0, 8, and 15. At day 22, Qa-1–FL9–specific CD8 T cells were detected by tets (Qa-1–FL9–PE and Qa-1–FL9–APC) within CD44^+^CD122^+^Ly49^+^ CD8 T cells. (**B**) TCR repertoire of Qa-1–FL9 Tet^+^ CD8 T cells. Single Qa-1–FL9–PE^+^ Qa-1–FL9–APC^+^ cells were sorted and subjected to sequencing for TCRα and TCRβ. Thirty-nine of TCRα and TCRβ pairs were analyzed based on their TCR V gene segments. Relative usage of TCRα and TCRβ V genes by these Tet^+^ single cells is depicted by donut charts. (**C**) TCR repertoire of Qa-1–Hsp60 Tet^+^ CD8 T cells. Single Qa-1–Hsp60–PE^+^ Qa-1–Hsp60–APC^+^ cells were sorted and sequenced for TCRα and TCRβ. Relative usage of TCRα and TCRβ V genes by these Tet^+^ single cells is depicted by donut chart. (**D**) Frequency and phenotype of Vα3.2^+^Vβ5^+^ cells within Ly49^+^ CD8 cells in the spleens and LNs of WT B6, Qa-1.D227K–KI and Qa-1–KO mice at 8 weeks of age (*n* = 6/group). (**E**) Qa-1–dependent differentiation of FL9 T cells: tet-mediated detection of TCR in 58C hybridoma transduced with FL9.2 and FL9.8 TCR (upper panel). Responsiveness of FL9.2-TCR– and FL9.8-TCR–expressing hybridoma upon stimulation with increasing dose of peptides measured by CD69 expression (lower panel). (**F**) Measurement of Qa-1–FL9 binding affinity of FL9.2 and FL9.8 TCR. FL9.2 TCR^+^ and FL9.8 TCR^+^ hybridoma were labelled with Qa-1–FL9–PE tets and incubated in the presence of anti-Qa-1 antibodies for the indicated time. Percentages of PE^+^ cells were measured at different time points as a measurement of tet dissociation level. (**G**) Tg TCR^+^ cells in TCR^+^ thymocytes and percent of active-Caspase–3^+^PD1^+^ cells in DP (CD4^+^CD8^+^) thymocytes in OT-I → WT B6, FL9.2 Tg → WT B6 BM chimera 8 weeks after BM reconstitution. (**H**) Ki67 and CD44 expression by OT-I and FL9.2 TCR Tg CD8^+^ T cells was measured as an indication of Ag encounter in the spleen and liver of OT-I → WT B6 and FL9.2 Tg → WT B6 BM chimera 8 weeks after BM reconstitution. OT-1 is also used as a control in Supplemental Figure 6B. *P < 0.05, **P < 0.01, ***P < 0.001, according to Wilcoxon-Mann-Whitney rank sum test.

We used Qa-1–FL9 and Qa-1–Hsp60 peptide tetramers to detect, sort, and analyze TCR expression by tetramer-positive (tet^+^) cells according to single-cell TCR sequencing (Figure 1A and Supplemental Table 1; supplemental material available online with this article; https://doi.org/10.1172/JCI170512DS1). Analysis of paired TCRs from 12 independent Qa-1–FL9 tet-binding cells revealed that 9 of 12 TCRs specific for Qa-1–FL9 tets expressed the TRAV9N3 gene encoding Vα3.2 and 9 of 12 expressed the TRBV12-1/2 genes encoding Vβ5.1,2 (Figure 1B and Supplemental Figure 1). Analysis of 11 independent Qa-1–Hsp60–specific CD8 T cells revealed that 8 of 11 also expressed TRAV9N3/Vα3.2, and 6 of 11 expressed the TRBV 12-1/2/Vβ5.1,2 (Figure 1C and Supplemental Figure 2). An additional analysis of control T cells that were not enriched by FL9-tet staining indicated that a potential bias introduced by PCR amplification was minimal and could not account for these findings (Supplemental Figure 3). Both TCR sets expressed nearly identical CDR1 and CDR2 sequences — which usually represent MHC contact elements — but carry distinct peptide-specific CDR3 regions. The TRAV9N3 and TRBV12-1/2 genes were essential for high affinity binding by FL9 T cells, since TCRs that did not express these paired genes displayed markedly reduced binding affinity to Qa-1–FL9 complexes (Supplemental Figures 1 and 5). Recognition of 2 structurally distinct self-peptides presented by Qa-1 by this conserved TCR repertoire suggests an interaction between highly conserved CDR1/CDR2 TCR regions and the class Ib Qa-1 molecule and with a second interaction between CDR3 and the FL9 and Hsp60 self-peptides.

We then asked whether Qa-1–restricted CD8 Tregs in non-Tg mice might also express the Vα3.2 and Vβ5 TCRs noted above. We found that triad^+^ (Ly49^+^CD122^+^CD44^+^) Vα3.2^+^/Vβ5.1,2^+^ CD8 Tregs were reduced by 60%–80% in mice carrying a Qa-1 deletion or Qa-1 D227K point mutation (Figure 1D and Supplemental Figure 4). In contrast, the numbers or percent of triad^+^ Vα3.2^–^/Vβ5.1,2^–^ CD8 T cells were not affected by either a Qa-1 deletion or mutation (Figure 1D). Moreover, residual triad^+^ Vα3.2^+^/Vβ5.1,2^+^ CD8 Tregs in Qa-1–KO mice displayed a 50%–75% reduction in the Ki67 proliferation marker (Figure 1D). Taken together, these findings suggest that Qa-1–restricted CD8 Tregs (Ly49^+^CD44^+^CD122^+^) are distinguished from MHC class Ia–restricted CD8 Tregs by expression of a conserved set of TCRs that are equipped to recognize pQa-1 complexed with structurally unrelated self-peptides.

### CD8 Treg phenotype of Qa-1–FL9–specific T cells.

To gain insight into the contribution of TCR usage to the differentiation and function of self-reactive CD8 Tregs, we cloned each of the 12 TCR pairs specific for Qa-1–FL9 into retroviral vectors and expressed them in 58C (TCR^–^) hybridoma cells. Expression of each TCR in 58C cells was accompanied by specific binding to Qa-1–FL9 but not Qa-1–Hsp60 tets (Supplemental Figure 5, A and B), presumably reflecting the peptide-specific CDR3 sequences noted above (Supplemental Figure 1). The binding avidity of each Qa-1–FL9–specific TCR was then defined according to a dose-response analysis of the concentration of FL9 peptide required for CD69 upregulation by each transduced hybridoma (Supplemental Figure 5C). A Qa-1–FL9–specific TCR with an intermediate (FL9.2) avidity and high avidity (FL9.8) for Qa-1–FL9 (Figure 1E) were defined further in an antigen dissociation assay, which confirmed the higher affinity of the FL9.8 TCR, as judged by increased retention of Qa-1–FL9 tet compared with the FL9.2 TCR (Figure 1F).

We generated Tg mice that express FL9.2 and FL9.8 self-peptide–specific TCRs using methods employed previously to generate OT-I TCR Tg mice that depended on insertion of pES.42.1c and pKS913.CB18.31 vectors (12). We then generated BM chimeras, which allow T cell development in a synchronized and homogenous environment, after reconstitution of lethally irradiated C57BL/6 (B6) hosts with BM hematopoietic cells from OT-I, FL9.2, or FL9.8 TCR Tg mice to study the contribution of these TCRs to the selection and development of CD8 Tregs. The percent of Tg TCR^+^ T cells in peripheral tissues was approximately 90% in the 3 BM chimeras that had been reconstituted with each TCR transgene (Figure 1G and Supplemental Figure 6A). Analysis of thymocytes revealed that approximately 20% of FL9.2 and 40% of FL9.8 thymocytes expressed markers of negative selection that included active caspase 3 and PD1, while OT-I thymocytes did not express these negative selection markers (Figure 1G and Supplemental Figure 6A). Analysis of peripheral T cells revealed that the 2 FL9 TCR transgenes, but not OT-I, displayed increased expression of CD44 and Ki67 (Figure 1H and Supplemental Figure 6B) and reduced levels of TCR and CD8, i.e., a CD8 T cell phenotype associated with chronic activation by self-antigens (13, 14) (Supplemental Figure 6C). Chronic exposure of CD8 T cells to self-antigen may also upregulate expression of NKG2D receptors (15, 16), which has been correlated with immunoregulatory function (17). FL9.8 T cells displayed an age-dependent upregulation of NKG2D expression (over 80% at 4 months), while FL9.2 T cells displayed a more modest increase (20%–40%) (Supplemental Figure 7, A and B).

**Figure 2:**
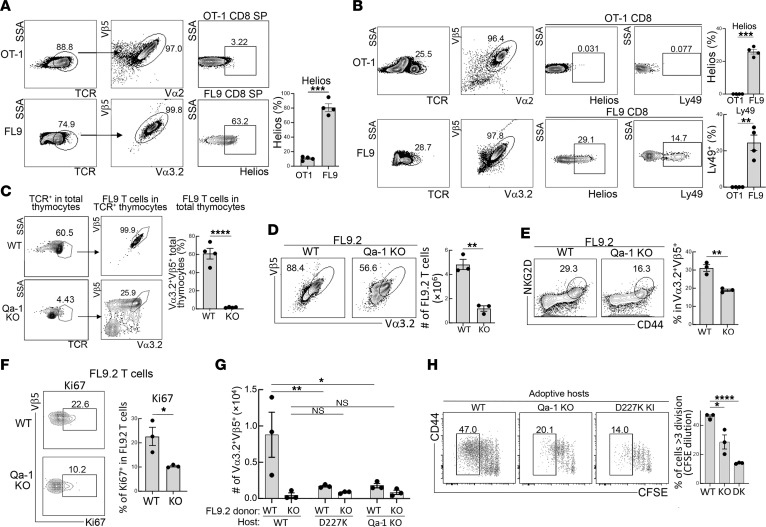
Qa-1–dependent differentiation of FL9 T cells. (**A**) Frequency of Tg TCR^+^ cells in the TCR^+^ thymic cells in OT-I– or FL9.2 TCR Tg mice (Va2^+^Vβ5^+^ for OT-I and Vα3.2^+^Vβ5^+^ for FL9 T cells) and levels of Helios expression. (**B**) Frequency of Tg TCR^+^ cells in the TCR^+^ splenic cells in OT-I or FL9 TCR Tg mice and levels of Helios and Ly49 expression. (**C**) Frequency of FL9 TCR Tg T cells in the TCR^+^ thymic cells in WT or Qa-1^–/–^.FL9 TCR Tg mice. Frequency of FL9 Tg T cells (Vα3.2^+^Vβ5^+^) in the total thymocytes is shown in graph (right). (**D**) Frequency of Vα3.2^+^Vβ5^+^ T cells in TCRβ^+^ spleen cells from FL9.2 TCR Tg mice on Qa-1–WT and –KO backgrounds (at 8 weeks old). Representative FACS plots for the detection of Vα3.2^+^Vβ5^+^ cells in spleen are shown (left panel). (**E**) Expression of CD44 and NKG2D by FL9.2 T cells in spleens of WT.FL9.2 TCR Tg and Qa-1^–/–^.FL9.2 TCR Tg mice. Representative FACS plots for NKG2D^+^CD44^+^ cells in the spleens of FL9.2 TCR Tg mice are shown on the left. (**F**) Expression of Ki67 by FL9.2 T cells in the LNs of WT.FL9.2 TCR Tg and Qa-1^–/–^.FL9.2 TCR Tg mice. (**G**) CFSE-labeled FL9.2 T cells developed in Qa-1–WT or Qa-1–KO mice were transferred into irradiated (800 rads) Qa-1–WT, Qa-1–KO and D227K-KI adoptive hosts. Seven days after transfer, Qa-1–WT or Qa-1–KO FL9.2 T cells were recovered from spleens of adoptive hosts. Numbers of FL9.2 T cells in the spleens of adoptive hosts are shown. (**H**) Percentage of Qa-1 WT FL9.2 T cells that undergo more than 3 divisions in Qa-1–WT, Qa-1–KO and D227K-KI hosts. Mean ± SEM is indicated. ****P < 0.0001, ***P < 0.001, **P < 0.01, *P < 0.05.

We then asked whether Tg expression of the FL9.2 TCR in the thymus instructed developing T cells to acquire and maintain the CD8 Treg phenotype (2). All TCR^+^ cells in the thymus express the TCR transgene at levels similar to developing OT-I thymocytes (Vα2, Vβ5), but only FL9.2 T cells expressed the canonical CD8 Treg transcription factor Helios (Figure 2A). After maturation, peripheral FL9.2 T cells maintained a characteristic Treg phenotype and expressed both Helios TF and Ly49 (Figure 2B). We then defined the contribution of pQa-1 recognition in Qa-1 WT and Qa-1 KO FL9.2 Tg mice. In the absence of the Qa-1 restriction element, FL9.2 T cells did not develop; only approximately 1% of thymocytes in the Qa-1 KO thymus expressed the FL9.2 TCR, compared with approximately 60% of thymocytes that expressed FL9 TCR in WT Tg mice (Figure 2C). This data suggests that Qa-1 represents the sole MHC restriction element for the FL9.2 TCR^+^ T cells rather than a crossreactivity of this TCR with other classical and nonclassical MHC molecules suggested previously (10, 18, 19). Deletion of the Qa-1 restriction element also resulted in an approximate 80% reduction in the numbers of both FL9.2 CD8 T cells (Figure 2D) and the higher affinity FL9.8 TCR^+^ T cells in the spleen (Supplemental Figure 8A). Residual FL9.2 TCR^+^ T cells expressed reduced levels of the CD44 and NKG2D receptors (Figure 2E and Supplemental Figure 8B) and the Ki67 proliferation marker (Figure 2F and Supplemental Figure 8C).

Although the numbers of FL9.2^+^ T cells were reduced by 70%–80% in mice that expressed a defective or deleted Qa-1, a substantial fraction remained. We asked whether these residual CD8 cells in the spleen and lymph node of Qa-1–deficient mice were functionally impaired. Transfer of residual FL9.2 T cells from Qa-1–KO mice into irradiated adoptive Qa-1–WT hosts revealed that very few (approximately 10%) survived, compared with the robust survival of FL9 T cells from Qa-1–WT donors (Figure 2G). We then asked whether recognition of Qa-1 in peripheral tissues was essential for continued survival of mature Qa-1–restricted FL9.2^+^ T cells that had initially differentiated in a Qa-1–sufficient (Qa-1–WT) environment. We found that FL9.2^+^ CD8 T cells from Qa-1–WT donors transferred into Qa-1–KO or D227K–knock-in (KI) hosts — who express a Qa-1 D227K point mutation that impairs the interaction between Qa-1 and the CD8 coreceptor — displayed poor survival, similar to that of CD8 TCR Tg cells transferred from Qa-1–deficient donors (Figure 2, G and H). These data suggest that Qa-1 expression is essential for both initial intrathymic development of Qa-1–restricted CD8 Treg and survival in peripheral lymphoid tissues.

### Detection and elimination of antigen-specific CD4 cells by Qa-1–restricted CD8 Treg.

**Figure 3:**
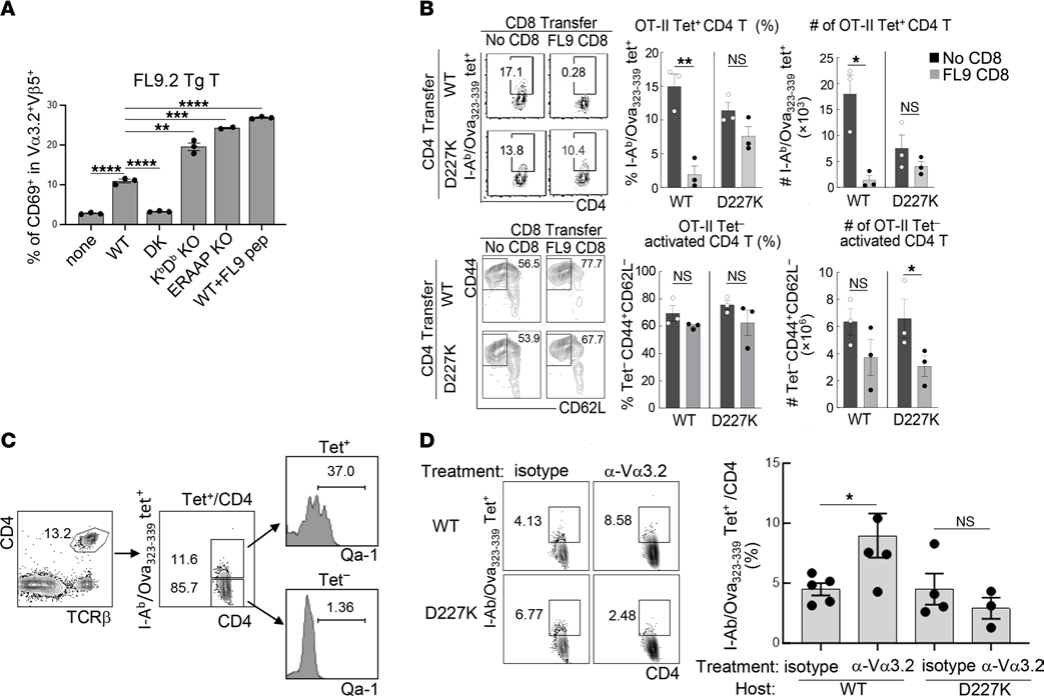
FL9 Tg CD8 T cells recognize and suppress activated CD4 T cells. (**A**) in vitro, conA-stimulated CD4 cells from WT B6, Qa-1.D227K–KI, KbDb-KO and ERAAP-KO mice were cocultured with FL9.2 T cells isolated from FL9.2 TCR Tg mice. WT CD4 cells loaded with the FL9 peptide were used as a positive control. After 20 hours, CD69 expression on FL9 Tg T cells were measured as a readout of TCR stimulation. (**B**) In vivo, WT or D227K mice were immunized with OT-II peptides in CFA. After 7 days, CD4 (CD4^+^CD25^–^) cells were isolated from immunized mice and transferred into WT B6 hosts with or without FL9 TCR Tg T cells followed by immunization with OT-II/CFA. Detection of I-A^b^/Ova_323-339_-specific CD4 T cells in the spleen of hosts by I-A^b^/Ova_323-339_ tets (upper). Percent and numbers of I-A^b^/Ova_323-339_ tet^+^ (upper) and I-A^b^/Ova_323-339_ tet^–^ activated (lower) CD4 cells recovered from adoptive hosts (middle and right). (**C**) Qa-1 expression by I-A^b^/Ova_323-339_ tet^+^ and tet^–^ CD4 cells. (**D**) WT B6 and D227K mice were immunized with Ova/CFA and injected with isotype or anti-Vα3.2 antibodies on day 0, before boosting on day 8 with Ova/IFA along with antibody injection. Frequency of I-Ab/Ova_323-339_ tet^+^ CD4 cells in blood were assessed on day 15. ****P < 0.0001, ***P < 0.001, **P < 0.01, *P < 0.05.

Although targeting of CD4 cells by CD8 Treg may reflect TCR-dependent recognition of pQa-1 complexes expressed by activated CD4 cells (2), the nature of these target complexes is not well understood. We asked whether expression of FL9-Qa–1 complexes by Ag-specific CD4 T cells represents a major functional target for CD8 Treg. We noted that FL9 TCR Tg T cells were efficiently stimulated by activated CD4 T cells from B6 (Qa-1–WT) mice but not by B6.Qa-1–D227K–KI mice (Figure 3A and Supplemental Figure 9A). Moreover, Kb^–/–^Db^–/–^ CD4 cells provoked stronger responses by FL9 TCR^+^ CD8 T cells, possibly reflecting the absence of a dominant Qdm default peptide derived from MHC class Ia that may competitively bind to Qa-1 (Figure 3A and Supplemental Figure 9A). Activated ERAAP-deficient CD4 cells strongly stimulated FL9 TCR Tg T cells, consistent with increased Qa-1–FL9 expression by cells lacking the ERAAP enzyme, which normally destroys this peptide (10) (Figure 3A and Supplemental Figure 9, A and B). Collectively, these findings suggest that activated CD4 cells express an ERAAP-sensitive Qa-1–FL9 ligand that is recognized by Qa-1–FL9–specific CD8 Treg.

We then analyzed this interaction in vivo. We tested the hypothesis that upregulation of Qa-1 by robustly activated CD4 T cells may depend on expression of high affinity TCRs (2, 20). To test this hypothesis, we characterized CD4 cells generated after immunization with OVA according to binding to OT-II peptide class II MHC tets, expression of Qa-1–FL9, and sensitivity to inhibition by CD8 Tregs. We transferred CD4 cells from OT-II–peptide immunized WT B6 or B6-D227K mice into B6 hosts with or without FL9.2 TCR Tg CD8 cells, followed by immunization with OT-II/CFA. Analysis of OT-II tet^+^ CD4 cells, which represent CD4 cells with the highest avidity for immunizing OVA, revealed that cotransfer of FL9.2 TCR Tg CD8 T cells inhibited more than 90% of OVA tet^+^ CD4 cells (Figure 3B, upper panel), while virtually all activated (CD44^+^CD62L^–^) CD4 cells that were tet^–^ were spared (Figure 3B, lower panel). Selective inhibition of relatively avid Ag-specific CD4 cells depended on Qa-1 targeting, since FL9 CD8 T cells suppressed the response of B6 (WT) CD4 T cells but not B6-D227K CD4 T cells (Figure 3B, upper panel). Moreover, analysis of Qa-1 expression by Tet^+^ and Tet^–^ CD4 T cells revealed that approximately 35% of activated Tet^+^ CD4 T cells expressed Qa-1, while less than 2% of Tet^–^ CD4 T cells expressed Qa-1 (Figure 3C). These data indicate that the Qa-1–FL9 peptide complex is expressed on a substantial proportion of CD4 cells after activation by Ag, and that CD4 cells expressing highly avid TCR (tet^+^) for cognate Ag strongly express Qa-1 (Qa-1^hi^) and are efficiently suppressed by Qa-1–restricted CD8 Treg in contrast to nonspecifically activated CD4 T cells.

Based on observations that Qa-1–restricted CD8 Tregs mainly express the Vα3.2/Vβ5 pair (Figure 1D), we asked whether Ab-mediated depletion of Vα3.2^+^ T cells after immunization to OVA might enhance the Ag-specific CD4 T cell responses. B6.WT, but not B6.Qa-1 D227K, mice immunized with OVA/CFA and boosted with OVA/IFA developed an increased frequency of OVA-specific CD4 cells (I-Ab/Ova_323-339_ Tet^+^) after depletion of Vα3.2^+^ T cells (Figure 3D). Virtually complete depletion of Vα3.2^+^ T cells could be achieved after anti-Vα3.2 Ab administration (Supplemental Figure 10). The marked increase in Qa-1 levels expressed by I-A^b^/Ova_323-339_ Tet^+^ CD4 cells confirms the above findings that CD8 Tregs selectively target antigen-specific CD4 cells (Figure 3C). These data also indicate that Qa-1–restricted CD8 cell targeting by anti-TCRα3.2 antibody might be employed to modulate CD8 Treg activity.

### Definition of FL9-superagonist peptides.

**Figure 4:**
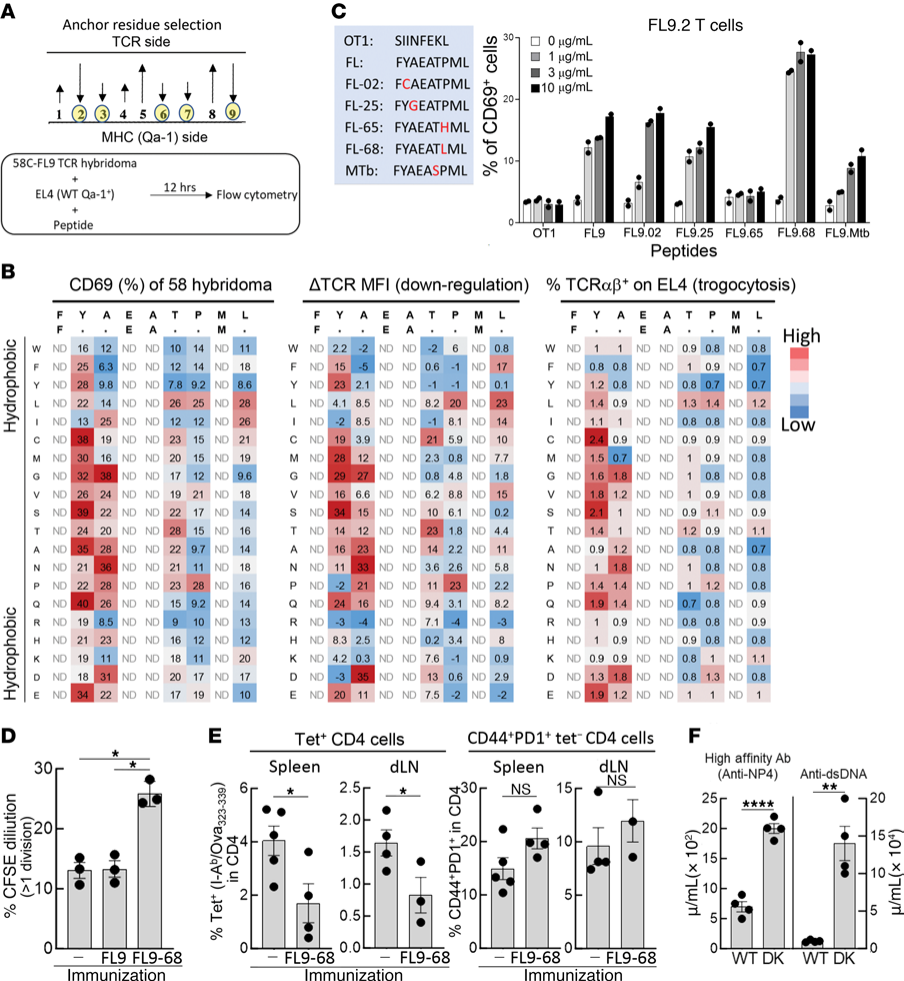
Identification of superagonists for FL9 T cells. (**A**) A library composed of 96 FL9 peptide variants (crude peptides) was generated by amino acid mutagenesis at the Qa-1 anchoring positions (p2, p3, p6, p7, and p9). FL9 TCR^+^ hybridomas were incubated with EL4 cells (Qa-1^+^) loaded with each FL9 peptide variant for 12 hours and CD69 expression and TCR downregulation were measured as an indication of TCR stimulation. (**B**) Activation of FL9 TCR^+^ 58C hybridoma after stimulation with FL9 peptide variants. CD69 expression by FL9 TCR^+^ 58C hybridoma after stimulation with each FL9 peptide variant (left). Downregulation of TCR is shown as ΔTCR MFI based on calculation 100–(Testing TCR MFI/Control TCR MFI) × 100 (%) (middle). Expression of Vα3.2 and Vβ5 on EL4 cells (trogocytosis) was measured (right). (**C**) Activation of FL9.2 T cells after stimulation with FL9 variants selected from library screen above. Dose-dependent activation of FL9 T cells was measured by culturing FL9.2 T cells with EL4 (Qa-1^+^) at various concentrations of indicated peptides (0, 1, 3, and 10 μg/mL). (**D**) CD45.1^+^ B6 hosts were adoptively transferred with FL9.2 T cells and immunized i.p. with PBS, FL9, or FL9-68 in CFA on day 0. After 6 days, proliferation of FL9.2 T cells (CD45.2^+^Vα3.2^+^Vβ5^+^) was measured by CFSE dilution (left). (**E**) CD45.1^+^ B6 mice that were vaccinated with FL9-68 in CFA or CFA alone on day 0 were immunized with Ova_323-339_ peptide in CFA on day 6. The frequency of I-A^b^/Ova_323-339_ Tet^+^ CD4 cells in activated (CD44^+^) CD4 cells was analyzed on day 14. (**F**) Comparison of high-affinity antibody and auto-antibody responses in WT and Qa-1.D227K mice. WT B6 and Qa-1.D227K.KI mice were immunized with NP_19_-KLH/CFA and boosted with NP_19_-KLH/IFA on day 10. High affinity anti-NP responses were measured on day 15. Levels of anti-dsDNA antibody were measured on day 21. Mean ± SEM is indicated. ****P < 0.0001, ***P < 0.001, **P < 0.01, *P < 0.05.

Since chronically activated CD4 cells express Qa-1–FL9 complexes, in vivo expansion of Qa-1–FL9–specific CD8 Tregs may promote elimination of these CD4 T cells in clinical settings. However, immunization of mice with the FL9 self-peptide did not elicit detectable expansion of CD8 Treg (Supplemental Figure 11), perhaps reflecting the relatively low Qa-1 binding affinity and consequent weak TCR activation by this self-peptide (2, 21, 22). We reasoned that efficient mobilization of Qa-1 restricted CD8 Tregs specific for self-peptides might require immunization with peptide analogs with increased Qa-1 binding activity. To systematically improve binding stability to Qa-1, we screened a peptide library composed of approximately 100 amino acid exchange variants of FL9 peptide at MHC-anchoring positions 2, 3, 6, 7, and 9 (Figure 4A). The FL9.2 TCR^+^ 58C hybridoma was incubated with EL4 (Qa-1^+^) cells that had been pulsed with FL9 peptide variants and monitored for CD69 upregulation and TCR downregulation to define the stimulatory activity of each peptide variant (Figure 4B, left and middle panels). The interaction of Qa-1–peptide complexes with the FL9.2 TCR was also evaluated by measurement of TCR trogocytosis, which reports the strength of TCR binding to defined pMHC ligands (23) (Figure 4B, right panel). Variant FL9 peptides that stimulated increased FL9.2 TCR^+^ hybridoma responses compared with the native FL9 peptide — as judged by CD69 expression, TCR downregulation and increased trogocytosis — were then subjected to dose-response analysis.

This analysis revealed that a FL9 peptide variant containing a P→L substitution at position 7 — termed FL9-68 — displayed markedly enhanced dose-dependent stimulatory activity for FL9.2 and FL9.8 TCRs compared with the cognate FL9 self-peptide (Figure 4C and Supplemental Figure 12). Immunization with the FL9-68 agonist peptide activated FL9.2 TCR^+^ CD8 T cells after transfer into congeneic (CD45.1^+^ B6) hosts or TCRα^–/–^ hosts compared with the native FL9 peptide (Figure 4D). Immunization of CD45.1^+^ B6 hosts with Ova_323-339_ in CFA followed by analysis of activated CD4 T cells revealed that FL9-68 vaccination inhibited the response of I-A^b^/Ova_323-339_ tet^+^ CD4 cells by more than 50% but did not reduce the numbers or percentage of nonspecifically activated CD4 cells that did not bind to the FL9-Qa–1 tet (Figure 4E). The finding that CD8 Tregs mainly target Tet^+^ CD4 cells (Qa-1^hi^) was consistent with the observation that defective CD8 Treg activity in Qa-1.D227K mutant mice resulted in a significant increase in high affinity Ab responses to immunizing antigens (NP-KLH) as well as anti-dsDNA antibodies (Figure 4F). Collectively, these findings suggest that FL9-specific CD8 Tregs preferentially suppress Qa-1^hi^ CD4 cells that express high avidity TCR for the immunizing antigen without generalized immune suppression and suggest that the FL9-68 peptide analog might be used to deliberately mobilize CD8 Tregs in clinical settings.

### Peptide-dependent mobilization of CD8 Tregs and inhibition of alloimmunity.

Antibody-mediated rejection (AMR) remains a major barrier to successful solid organ transplantation (24). The observation that CD8 Tregs mainly target high affinity CD4 cells suggested that mobilization of CD8 Tregs may allow suppression of robust responses to MHC-disparate tissues without generalized immune suppression and the potential risk of increased vulnerability to pathogenic infection. Since pathogenic alloantibodies mediating AMR are produced mainly by germinal center (GC) B cells after induction by Tfh cells (25), increased expression of the Qa-1–FL9 complex by Tfh cells activated by alloantigens may allow targeting and suppression of pathogenic CD4 cells by Ag-specific CD8 Tregs. Our recent analysis of the allograft response of B6.Qa-1–mutant (B6.Qa-1–D227K) mice indicated that disruption of the interaction between CD8 Treg and Qa-1 in recipients of a fully allogenic heart transplant model results in unchecked Tfh cell proliferation and accelerated Ab-mediated allograft injury (26).

**Figure 5:**
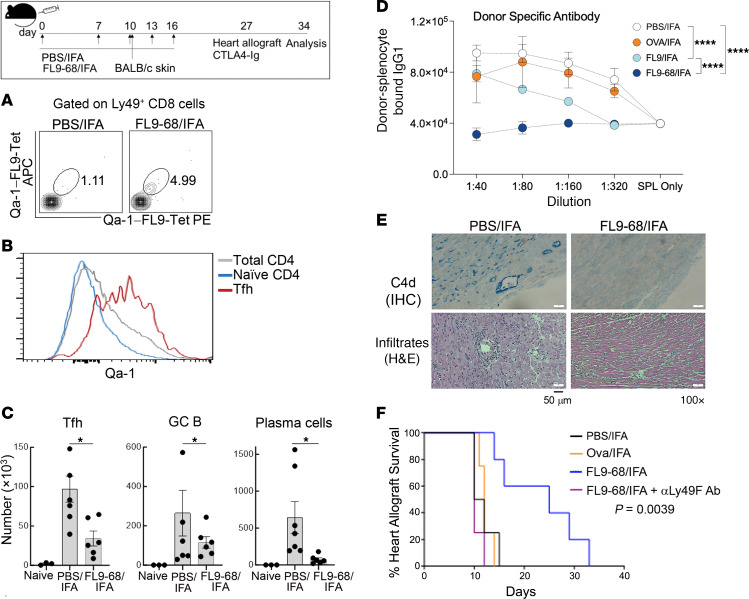
Superagonist peptide vaccination inhibits AMR in heart transplantation. (top panel) Schematic of experimental design. B6 mice were vaccinated with FL9-68 peptide in IFA or PBS in IFA on the indicated days, followed by sensitization with BALB/c skin on day 10. Mice were further vaccinated with FL9-68 in IFA or PBS/IFA on days 10, 13, and 16. On day 27, BALB/c hearts were heterotopically transplanted to the abdominal cavity of B6 recipients. 250 μg of CTLA-4 Ig was administered i.v. after transplantation and recipients were analyzed on day 34. (**A**) Frequency of Qa-1-FL9-Tet^+^ CD8 (CD44^+^CD122^+^Ly49^+^) T cells in dLN of IFA- or FL9-68/IFA-vaccinated B6 hosts is shown. (**B**) Qa-1 expression by total, naive CD4 and Tfh cells in the draining lymph node (dLN) of skin-sensitized B6 hosts after BALB/c heart graft. (**C**) Numbers of Tfh, GC B and plasma cells in dLNs of naive B6 mice or PBS/IFA- or FL9-68/IFA-vaccinated B6 recipients. (**D**) Donor-specific antibodies (IgG1) in naive mice or PBS/IFA-, OT-I/IFA-, FL9/IFA- or FL9-68/IFA–vaccinated B6 recipients of skin grafts were measured in the serum collected on day 26, the day before heart transplantation. BALB/c donor splenocytes were incubated with serially diluted serum followed by detection with fluorescence-labeled anti-mouse IgG1 antibodies. Statistical analysis was performed with 2-way Anova involving mixed-effect analysis. (**E**) C4d deposition (blue) in heart allografts (upper panel). Tissue sections from heart grafts of B6 mice that were vaccinated with PBS/IFA alone or FL9-68/IFA were stained with anti-C4d antibodies. H&E stain of heart allograft showing graft infiltrating lymphocytes (lower panel). (**F**) Heart graft survival in mice vaccinated with FL9-68/IFA, FL9-68/IFA with α-Ly49F Ab, an irrelevant peptide, Ova/IFA, and PBS/IFA alone as control. Median Survival (days): FL9-68/IFA, 25; FL9-68/IFA with α-Ly49F Ab, 10; Ova/IFA, 12; and IFA alone, 11. Mean ± SEM is indicated. ****P < 0.0001, ***P < 0.001, **P < 0.01, *P < 0.05.

To test the ability of FL9-specific CD8 Tregs to inhibit humoral allograft rejection, we first asked whether expansion of FL9-specific T cells by the FL9-68 peptide might suppress the antiheart graft response (27). We transplanted BALB/c (haplotype: H-2^d^) heart allografts into previously BALB/c skin allograft–sensitized B6 (H-2^b^) hosts, since heart transplants into nonsensitized B6 hosts induce strong cellular rejection but only weak humoral responses (28). B6 mice vaccinated with FL9-68 peptide during the skin allograft sensitization displayed a 4–5-fold increase of Ly49F^+^ FL9-specific CD8 cells (Figure 5A).

Seventeen days after skin transplantation with or without FL9-68 vaccination, alloantigen (skin) sensitized B6 hosts were transplanted with BALB/c heart allografts, which are rejected by a robust antigraft antibody response. We observed significant upregulation of Qa-1 expression by Tfh cells in draining lymph nodes (dLN) compared with other CD4 T cell subsets at day 7 after heart transplantation in these skin allograft–sensitized B6 hosts (Figure 5B). Hosts that had been vaccinated with FL9-68/IFA displayed a significant reduction of GC responses in the dLN, along with reduced numbers of Tfh cells (PD-1^+^CXCR5^+^CD4^+^), activated GC B cells (FAS^+^GL-7^+^B220^+^), and plasma cells (B220^–^CD138^+^) compared with the control group injected with PBS/IFA (Figure 5C). We also observed a vaccine-dependent reduction in the titers of donor-specific antibody (DSA). Vaccination with FL9-68 had a notably superior effect in suppression of DSA responses compared with the native FL9 peptide (Figure 5D). Decreased germinal center activity and DSA levels were associated with amelioration of graft pathology, as measured by reduced levels of C4d deposition and immune cell infiltration (Figure 5E). The major impact of FL9-68 peptide vaccination was a significant prolongation of heart graft survival time compared with vaccination with an irrelevant peptide (Ova) and adjuvant (Addavax) administration (Figure 5F). Finally, the protective effect of FL9-68 peptide was reversible when CD8 Tregs were depleted with α-Ly49F Ab (Figure 5F).

**Figure 6:**
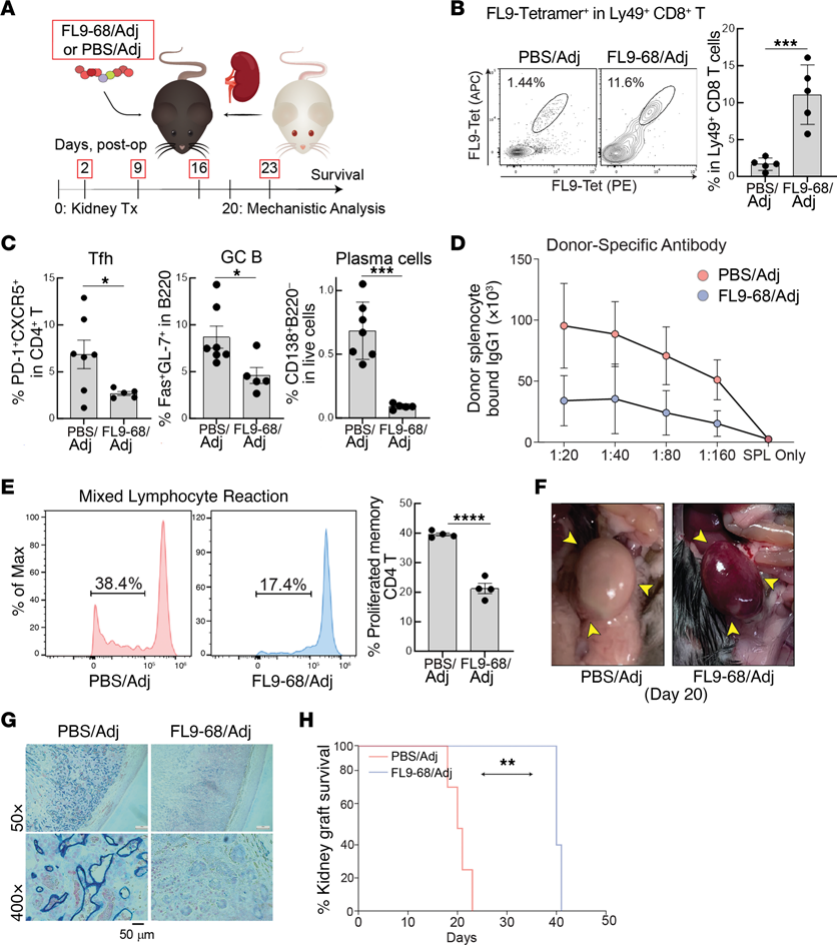
FL9-68 ameliorates AMR and prolongs graft survival in kidneys. (**A**) Schematic of experimental design. (**B**) Frequency of FL9-Qa–1–specific CD8 (CD44^+^CD122^+^Ly49^+^) T cells in mice with or without FL9-68 immunization (day 20 after transplant). (**C**) Frequency of Tfh cells (PD-1^+^CXCR5^+^CD4^+^), activated GC B cells (FAS^+^GL-7^+^B220^+^), and plasma cells (B220^–^CD138^+^) in the graft recipients with or without FL9-68 immunization. (**D**) Donor-specific antibodies (IgG1) in PBS/ Adj- or FL9-68/ Adj-vaccinated B6 recipients of kidney grafts. (**E**) Proliferation of activated CD4^+^ T cells from PBS/Adj- or FL9-68/Adj-immunized recipients, when cocultured with irradiated donor (BALB/c) splenocytes. Mixed lymphocyte reaction, comparison of the proliferative response of memory (CD44^+^) CD4 T cells isolated from the indicated group of kidney allograft recipients at day 20 upon exvivo stimulation with irradiated donor (BALB/c) splenocytes, as judged by CellTrace Violet on day 3 of coculture. (**F**) Gross anatomy of kidney allograft at day 20. (**G**) IHC for C4d deposition (blue). (**H**) Survival of kidney allograft measured by survival of recipients with absence of native kidney function. Median Survival (days): FL9-68/Adj, 40; PBS/Adj, 20.5. Mean + SEM is indicated. ***P < 0.001, **P < 0.01, *P < 0.05.

The effect of peptide-mediated expansion of CD8 Tregs on antiallograft immunity against fully mismatched kidney transplants was also tested (Figure 6A). Here, rejection depends on a combined cellular- and antibody-mediated response (28). Analysis of allograft-dLN on day 20 following kidney transplantation revealed a more-than 7-fold increase in FL9-specific Tregs from FL9-68–vaccinated mice, as measured by FL9-Qa–1 Tet^+^ Ly49^+^ CD8 T cells (Figure 6B). Similar to observations in the sensitized heart transplant model, administration of FL9-68–Adj but not adjuvant (Addavax) alone markedly reduced GC responses, as judged by reduced frequency of Tfh cells, activated GC B cells, and plasma cells (Figure 6C), as well as a significant decrease in DSA (Figure 6D). Ex vivo stimulation of celltrace violet–stained memory (CD44^+^) CD4^+^ T cells from B6 (H-2^b^) hosts with irradiated donor BALB/c (H-2^d^) lymphocytes also revealed that FL9-68 vaccination reduced allospecific CD4 T cell memory responses in FL9-68 vaccinated hosts (Figure 6E). These vaccine-induced changes were accompanied by reduction in the antibody-mediated allograft response, as judged by reduced C4d deposition in the peritubular capillaries of kidney allografts (Figure 6, F and G) and substantial prolongation of allograft survival compared with controls (Figure 6H).

Collectively, these data indicate that peptide-based mobilization of CD8 Tregs that recognize pathogenic CD4 Tfh cells represents a promising therapeutic approach for drug-free reduction of Ab-mediated injury in a murine model of heart and kidney allografts.

## Discussion

There is increasing evidence that suppression of pathogenic host responses by CD8 Tregs depends on precise recognition of MHC class 1b-self–peptide complexes expressed by activated CD4 effector cells (2, 6, 29). However, the basis for this recognition and targeting of chronically activated CD4 T cells has been obscure. Our characterization of TCRs expressed by Qa-1–restricted CD8 Tregs revealed a surprisingly restricted expression of CDR1/CDR2 regions expressed by both TCRα and β chains. This interaction may allow Qa-1–restricted CD8 T cells specific for self-peptides to survive peptide-mediated negative selection in the thymus and to recognize activated CD4 T cells that upregulate Qa-1 after recognition of antigen by high affinity TCRs. Analysis of the development of immature thymocytes that expressed a Vα3.2/Vβ5 TCR transgene specific for Qa-1-FL9 self-peptide indicated that acquisition of the CD8 Treg phenotype in the thymus and periphery depended on expression of Qa-1 in the thymus and peripheral tissues. Indeed, the residual populations of FL9 TCR^+^ CD8 T cells that persisted in the absence of Qa-1 displayed markedly reduced survival and impaired activation in adoptive environments that expressed a WT Qa-1 phenotype (Figure 2, G and H). Analysis of the Qa-1–restricted CD8 Treg subpopulation within CD8 T cells also revealed that the restricted Vα3.2/Vβ5 TCR pairing depended on expression of Qa-1 by host tissues.

Previous studies have indicated that the functional differentiation of CD8 Tregs is restricted by the MHC class Ib molecule Qa-1. The B6.Qa-1-D227K mutant harbors an amino acid exchange mutation that disrupts binding of Qa-1 to TCR/CD8 receptors expressed by CD8 T cells. These mutant mice develop a lupus-like autoimmune disease secondary to impaired CD8 Treg activity (30). In the present study (e.g., Figure 2), we investigated the contribution of Qa-1 to the development of FL9 T cells. Our results clearly demonstrate that functional FL9 T cells failed to develop in the absence of a functional Qa-1 restriction element (Figure 2C). The residual FL9 T cells that appeared in peripheral tissues in the absence of Qa-1 displayed significantly reduced levels of activation and proliferation, and transfer of these cells into Qa-1 WT hosts did not restore their functional properties. Moreover, FL9 T cells that developed in Qa-1 WT mice required the continuous presence of Qa-1 for proliferation, and their viability and function were substantially impaired in both Qa-1–KO and Qa-1-D227K adoptive hosts. Collectively, these findings support the view that Qa-1 serves as the critical restriction element for the development of functional FL9 T cells.

MHC-E–restricted unconventional CD8 T cells have been shown to develop into both effector and regulatory lineages (2, 31, 32). Our findings suggest that expression of a narrow and defined TCR repertoire may be a decisive event in guiding immature CD8 thymocytes into a regulatory, rather than an effector, CD8 T cell lineage. Our findings suggest that MHC-E–restricted CD8 T cells that express TCRs that recognize self-peptides may differentiate into mature CD8 T cells that express canonical features of Tregs, including Helios and Ly49 as well as a central memory phenotype, reflecting their continuous recognition of self-antigen. Definition of the canonical TCR pairs expressed by CD8 Tregs also opens the possibility of selective activation or deletion of these MHC-E–restricted CD8 Tregs by antibodies specific for these TCRs and modulation of their activity in pathologic conditions that include autoimmune diseases and cancers.

The TCR-based recognition of pQa-1 by CD8 Tregs may also account for precise elimination of CD4 T cells that express high avidity TCRs for cognate Ag. Elevated expression of the Qa-1-FL9 complex by specifically activated CD4 cells may allow selective monitoring of Ag-activated but not nonspecifically activated CD4 T cells by CD8 Tregs. Thus, more than 90% of the high affinity tet^+^ CD4 T cell population was eliminated by CD8 Tregs, while nonspecifically activated but tet^–^ CD4 cells were spared (Figure 3). Robust upregulation of Qa-1 by tet^+^ CD4 T cells may also allow efficient targeting of Tfh cells, which are the major cellular source of helper function, without generalized immune suppression (20, 33).

CD8 Tregs expressed relatively low levels of CD8 and TCR, reflecting their self reactivity (Supplemental Figure 6C), and may be significantly more anergic than T cells specific for foreign antigens. These considerations suggested that mobilization of CD8 Tregs required identification of self-peptide variants with increased agonistic activity (34, 35). We screened synthetic FL9 peptides containing altered residues at Qa-1 anchoring positions to identify potential agonists with enhanced binding to pMHCI and increased immunogenicity. The FL9-68 peptide variant, which includes a P→L amino acid exchange at position 7, displayed significantly enhanced stimulatory activity in vitro and in vivo. Activation of CD8 Tregs by FL9-68 peptide agonist resulted in CD8 Treg expansion, inhibition of Tfh cell and GC B cell responses and reduced production of antigraft antibodies in mouse models of heart and kidney transplantation, resulting in significantly prolonged organ graft survival. Conversely, depletion of CD8 Treg with α-Ly49F Ab abrogated the impact of FL9-68 peptide vaccination (Figures 5 and 6).

Multiple autoimmune diseases have been associated with autoantibody generation secondary to dysregulated high affinity Tfh expansion (5, 36, 37) and CD8 Treg-mediated control of autoantibody generation may also be an essential mechanism to inhibit autoimmune disease development (2). Moreover, uncontrolled Tfh cell proliferation is also a prominent feature of antibody-mediated allograft rejection, a leading cause of long-term graft failure that currently lacks an effective therapeutic strategy (24). We show here that CD8 Tregs can be expanded and activated in a pMHC-specific fashion to efficiently target CD4 Th cells with high affinity for cognate antigen, including self-antigens as well as allo antigens. While we have used a peptide-dependent strategy to mobilize CD8 Tregs, characteristic expression of TCR Vα/Vβ by CD8 Tregs might also be exploited via anti-TRAV antibodies (targeting conserved CDR1/CDR2) to mobilize CD8 Tregs to efficiently inhibit or eliminate pQa-1^hi^ pathogenic CD4 cells. The efficacy of CD8 Treg expansion followed by inhibition of autoantibody generation and accompanying pathology can be tested in mouse models of autoimmune disease, including EAE, T1D (NOD) and SLE (BXSB-Yaa), or in various alloimmune models as shown above. Identification of human TCR homologous to the murine Vα3.2/Vβ5.1 TCR set (7) may allow their selective mobilization or depletion and form the basis of novel and effective treatments for disorders that reflect excessive or dysregulated immune responses.

While the immune function, phenotype, and genetic program of Qa-1-restricted CD8 Tregs have been studied extensively over the last decades, more recent studies (6, 7) have revealed the existence of a subset of CD8 Tregs that also recognize peptides in the context of MHC class Ia. For example, Saligrama et al. (6) observed coordinated expansion of CD8 T cells along with antigen-specific CD4 cells during EAE progression, including expansion of a CD8 T cell subset that suppressed antigen-specific CD4 T cells (despite lacking specificity for the immunizing MOG antigen). Extensive library screening identified surrogate peptides presented by MHC class Ia that expand CD8 Tregs upon immunization. However, the corresponding endogenous peptides displayed by activated CD4 T cells remained unidentified. Interestingly, the CD8 Tregs described in this study also expressed the triad of surface markers: CD44, CD122, and Ly49 characteristic of CD8 Tregs (2).

Identification of the Ly49 surface marker expressed by murine CD8 Tregs has also led to the recent definition of human CD8 Tregs that express KIRs, the human functional homologs of murine Ly49. Human CD8 Tregs also express inhibitory subtypes of KIR (KIR3DL1 and KIR3DL2/L3), similar to the inhibitory murine Ly49 subtypes expressed by murine CD8 Tregs (7). Suppressive activity entails recognition of peptides presented by HLA-A and HLA-E molecules expressed by pathogenic autoreactive CD4 cells. TCR analysis of KIR^+^ CD8 T cells compared with KIR^–^ CD8 T cells revealed that the TCRs expressed by KIR^+^ CD8 T cells have a more restricted TCR repertoire (7). Since KIR^+^ CD8 T cells from healthy donors and patients with distinct autoimmune disease could be grouped together based on the similarity of their expressed TCR repertoire, these cells may recognize common antigens expressed by CD4 cells that are activated by particular pathological conditions. Translation of our findings regarding preferential usage of Vα3.2 by murine CD8 Tregs requires identification of the homologous and dominant human TCR, since several studies have suggested homology between most mouse TCR V families and human TCR V families (38). Single cell-Seq of TCRs from HLA-E-FL9 tet^+^ and HLA-E-Hsp60 tet^+^ CD8 T cells may facilitate the application of human anti-Vα(X) treatment for activation or depletion of antigen-specific CD8 Tregs.

## Methods

### Mouse models.

C57BL/6 (B6), B6.SJL-Ptprc^a^Pepc^b^/BoyJ (B6.CD45.1), BALB/cJ, C57BL/6-Tg(TcraTcrb)1100Mjb/J (OT-1), B6.129P2-H2-K1^tm1Bpe^H2-D1^tm1Bpe^/DcrJ (Kb^–/–^Db^–/–^), and B6.129S2-TCRa^tm1Mom^/J (TCRα^–/–^) mice were obtained from the Jackson laboratory. B6.Qa-1.D227K KI and B6.Qa-1^–/–^ (B6.129S6-H2-T23^tm1Cant^/J) mice were generated in the laboratory and backcrossed for more than 10 generations, as previously described (29, 39, 40). B6.FL9.2 TCR Tg and B6.FL9.8 TCR Tg mice were generated in the laboratory, as described below, and maintained on Qa-1–WT and –KO backgrounds. B6.ERAAP^–/–^ mice were provided by Kenneth Rock (UMASS Medical Center, Worcester, Massachusetts, USA). All experiments were performed with age- (6–8 week old mice, unless otherwise indicated) and sex-matched mice.

### Antibodies and flow cytometry.

Fluorescence-labeled antibodies for B220 (RA3-6B2), CD3ε (17A2), CD4 (RM4-5), CD69 (H1.2F3), CD8α (53-6.7), CD8β (YTS156.7.7), Fas (SA367H8), Ki67 (16A.8), NKG2D (A10), PD-1 (29F.1A12), TCRβ (clone: H57-597), Vα2 (B20.1), Vα3.2 (RR3-16), and Vβ5.1/5.2 (MR9-4) were purchased from Biolegend; CD122 (TM-β1), CD44 (IM7), CXCR5 (SPRCL5), FoxP3 (FJK-16s) and Ly49C/I/F/H (14B11) from eBioscience and NKG2A (20d5) and Qa-1b (6A8.6F10.1A6) from BD Biosciences; and active Caspase 3 (5A1E) from Signaling Technologies. For the detection of FL9 T cells, Qa-1b/FL9-PE, Qa-1b/FL9-APC, Qa-1b/Hsp60p216-PE, and Qa-1b/Hsp60p216-APC tets were generated by NIH tetramer core facility and provided for this study. I-A^b^/Ova_323-339_ tets were purchased from MBL International.

### Measurement of antibody responses in WT and D227K mice.

WT B6 and D227K mice were i.p. immunized with NP19-KLH in complete Freund’s adjuvant (CFA) and boosted with NP19-KLH in incomplete Freund’s adjuvant (IFA) on day 10. Serum samples were collected from these mice on day 15. To measure high affinity anti-NP antibodies, ELISA plates were coated with 0.5 μg/mL NP4-BSA (Biosearch Technologies). A serum sample collected 14 days after immunization with NP19-KLH in CFA, along with reimmunization with NP19-KLH in IFA, was used as a standard. This immune serum was diluted at 1:4,000, which was defined as 100 units/mL. High affinity antibodies were measured by incubating plates with biotinylated anti-mouse IgG, followed by streptavidin peroxidase.

### Identification and isolation of FL9-specific TCRs.

Bone marrow–derived DCs were generated from Kb^–/–^Db^–/–^ mice in the presence of 20 ng/mL GM-CSF. After 6 days, DCs were stimulated with 50 ng/mL LPS for 12 hours. DCs were irradiated (30 Gy) and pulsed with FL9 peptide by incubating with 10 μg/mL FL9 peptides for 2 hours at 37º C. FL9-loaded Kb^–/–^Db^–/–^ DCs were injected into WT B6 mice at days 0, 8, and 15. At day 22, Qa-1b/FL9 tet^+^ cells were detected in the CD44^+^CD122^+^Ly49^+^ CD8 subset and single tet^+^ cells sorted by FACS. Identification of TCRα and TCRβ chains for each sorted cells was performed according to a previously published protocol (41). In brief, 1-step reverse transcription–PCR (RT-PCR) was performed by adding RT-PCR mix to each well. Primers for the RT-PCR mix include the leader sequences and constant region sequences of TCRs where adapter sequences were added to the 5′ end of the leader primers (41) (Supplemental Table 1). cDNA from this RT-PCR was used to amplify TCRα and TCRβ separately using the nested PCR principle. The PCR products were sequenced using mTRAC_1st2R and mTRBC_1st2R primers for TCRα and TCRβ amplicons, respectively, and analyzed with the IMGT/V-Quest algorithm (http://www.imgt.org).

### Generation of TCR^+^ hybridoma and TCR affinity test.

The cDNAs encoding the TCRα and TCRβ chain were inserted into the pMIG vector that contains GFP cassette, which was transfected into the PLAT-E cells using FuGENE6 (Promega). The culture medium was replaced with the fresh medium in 24 hours and supernatant was collected 72 hours after transfection and used to transduce TCR^–/–^ 58C hybridoma. Expression of TCRα and TCRβ pairs on the surface of 58C hybridoma was analyzed by staining with Qa-1b-FL9 tets, anti-CD3ε, and anti-TCR Vβ antibodies.

Relative affinity of FL9.8 and FL9.2 TCRs was analyzed by measuring the tet staining decay kinetics (42). FL9.8 TCR^+^ and FL9.2 TCR^+^ hybriboma were incubated with PE-conjugated Qa-1b/FL9 tets in the presence of anti-Qa-1 antibodies. Cells were fixed at different time points (0–120 minutes) after initiation of incubation, and the intensity of PE staining was measured as an indication of tet binding by flow cytometry.

### Generation of FL9 TCR Tg mice.

FL9.2 and FL9.8 TCR transgenes were generated by replacing the TCR V(D)J elements of the pES.42.1c and pKS913.CB18.31 vectors, which have been used previously to generate OT-I TCR Tg mice (12), with each TCRα and TCRβ cDNA fragments for FL9.2 and FL9.8 TCRs. The vector was linearized and used to target C57BL/6 ES cells using standard methods at the Transgenic Core Facility at Beth Israel Deaconess Medical Center (Boston, Massachusetts, USA). Founder lines for the FL9.2 and FL9.8 TCR Tg mice were established after genotyping with the following primers: common primer set for both FL9.2 and FL9.8 TCRα 5′- CTAGAAGACTCAGGGTCTGA-3′ and 5′- TCGGCACATTGATTTGGGAGTCA-3′ amplified 1 kbp for the transgene; a primer set for FL9.2 TCRβ 5′- ACACTGTCCTCGCTGATTCTG-3′ and 5’- GATGTGAATCTTACCGAGAACAGTCAGTCTGGTTC-3′; and a primer set for FL9.8 TCRβ 5′- TAACACTGTCCTCGCTGAC-3′ and ATACAGCGTTTCTGCACTAG-3′, both amplified 500 bp for transgene.

### Peptide mutagenesis and superagonist peptide screen.

A peptide library was generated by single mutation of each Qa-1 anchoring position (p 2, 3, 6, 7, and 9) of the FL9 peptide (FYAEATPML) with 20 aa, which is composed of 96 FL9 variant peptides. FL9 TCR^+^ 58C hybridomas were incubated with EL4 cells that were pulsed with each FL9 variant. After 12 hours, CD69 expression and levels of TCR expression were measured by flow cytometry. For analysis of the binding strength of FL9 TCR with Qa-1-FL9 variants, trogocytosis was measured directly by the detection of FL9 TCR (Vα3.2^+^Vβ5^+^) on EL4 cells. FL9 TCR^+^ 58C hybridomas were cocultured with EL4 cells that were pulsed with FL9 variant peptides from the library. After 2 hours, the percentage of Vα3.2^+^Vβ5^+^ EL4 cells was assessed by flow cytometry as a measurement of trogocytosis.

### Adoptive transfer and in vivo suppression assay.

B6.Qa-1.WT or B6.Qa-1.D227K–KI mice were immunized i.p. with 100 μg Ova_323-339_ peptide in CFA. After 7 days, 1 × 10^5^ CD25^–^ CD4 cells were isolated from these mice and transferred into WT B6 mice along with FL9 Tg T cells. WT B6 adoptive hosts were immunized on the footpad with 20 μg Ova_323-339_ peptide in CFA. After 7 days, the frequency and numbers of I-A^b^/Ova_323-339_ tet^+^ CD4 cells and activated CD4 cells were assessed in the inguinal and popliteal LNs of B6 hosts by flow cytometry.

### FL9 peptide immunization.

CD45.1^+^ B6 or TCRα^–/–^ mice were transferred with CFSE-labeled 2 × 10^6^ FL9.2 Tg T cells followed by i.p. immunization with 100 μg FL9 or FL9-68 peptides in CFA. Proliferation and activation of FL9.2 Tg T cells in the adoptive hosts were analyzed by assessing CFSE dilution, Ki67, and CD69 expression at days 3 and 6 after transfer.

### Heart transplantation of skin-sensitized hosts and analysis of immune response.

B6 mice were immunized i.p. with 50 μg FL9-68/IFA or PBS/IFA at days 0 and 7 followed by a BALB/C → B6 skin transplant at day 10. FL9-68/IFA or PBS/IFA immunization was repeated on days 10, 13, and 16. An additional group of hosts were treated with 30 μg of α-Ly49F Ab (Novus Biologicals, clone HBF-719) on days of immunization to deplete CD8 Tregs. At day 27, fully vascularized BALB/c hearts were transplanted into the abdominal cavity of B6 mice using microsurgical techniques, as previously described (43). Heart graft survival was determined by monitoring palpable heart beating. At day 16 after skin sensitization, levels of FL9 T cells, Tfh, GC B, and plasma cells in dLNs were analyzed by flow cytometry. Serum was collected from the heart graft recipient B6 mice that were either immunized with FL9-68/adjuvant or adjuvant alone at day 16. Serially diluted serum was incubated with 1 × 10^6^ donor splenocytes in total volume 100 μL PBS for 30 minutes followed by detection of surface-bound antibodies on CD4 cells using anti-CD4 (Biolegend, Clone RM4-5) and anti-mouse IgG1 antibodies (BD Biosciences, Clone A85-1). Histological analysis of heart grafts was performed by InvivoEx company using anti-C4d Ab (Hycult Biotech) and Vector Blue Alkaline Phosphatase Substrate Kit (Vector Laboratories).

### Kidney transplantation and analysis of immune response.

The left kidney of BALB/c mice (H-2^d^) was recovered using a full-length ureter and transplanted into a B6 host (H-2^b^). The ureter of the remaining native kidney was then ligated on postoperative day 2–4 to inhibit native kidney function. Surgical success was determined if mice survived 7 days postsurgery (POD). Transplanted B6 hosts were treated i.p. with FL9-68 (50 μg) or PBS emulsified in adjuvant (Addavax, InvivoGen), once a week starting POD2. On day 20 following kidney transplantation (*n* = 5–7/group), allograft draining lymphoid tissues were assessed for FL9-specific Tregs (Qa-1-FL9 Tet^+^), Tfh, GC B, and plasma cells, DSA levels in sera, capillary C4d deposition, and gross anatomy of kidney allografts. Survival of kidney allografts was measured by survival of recipients with absence of native kidney function.

For the mixed lymphocyte reactions in the heart and kidney models, CD25^–^ CD4 T cells were isolated from dLN of B6 hosts and cocultured with irradiated BALB/c donor splenocytes. Proliferation was measured by immunofluorescence with Celltrace violet.

### Statistics.

Prism v.9.0 (GraphPad Software) was used for statistical analyses. Statistical significance was calculated according to Wilcoxon-Mann-Whitney rank sum test for comparison of 2 conditions; Kruskal-Wallis test was performed for comparison of more than 2 conditions. A *P* value of < 0.05 was considered to be statistically significant.

### Study approval.

All research with animal models was subject to prior review and approval and conducted in compliance with institutional guidelines set forth by the Animal Care and Use Committees of the Dana-Farber Cancer Institute and the Brigham & Women’s Hospital.

### Data availability.

Data are available upon reasonable request from the corresponding authors subject to institutional review and approval. Values for all data points in graphs are reported in the Supporting Data Values file.

## Author contributions

HK, HN, JYC, JRA, and HC conceptualized the project. HK, HN, JYC, JRA, and HC developed the methodology. HK, HN, JYC, XC, AD, QL, AEW, HZ, AS, ZS, and CD performed the experiments. HK, HN, JYC, XC, AD, QL, AEW, HZ, AS, ZS, and CD were responsible for visualization. JRA and HC acquired funding. JRA and HC were the project administrators. HK, JRA, and HC were responsible for supervision. HK and HC wrote the original draft of the manuscript. All authors were responsible for reviewing and editing the manuscript. Equal contribution author order was determined by the authors themselves.

## Supplementary Material

Supplemental data

Supporting data values

## Version 1 11/07/2023

In-Press Preview

## Version 2 01/02/2024

Electronic publication
