# Supplementary material for: A narrow T cell receptor repertoire instructs thymic differentiation of MHC class Ib–restricted CD8+ regulatory T cells
Source: J Clin Invest. 2024 Jan 2;134(1):e170512. doi: 10.1172/JCI170512 (PMC10760956; doi:10.1172/JCI170512)
Supplement: Supplemental data [file jci-134-170512-s131.pdf]

Supplemental Figure 1. TCR repertoire of Qa-1–FL9 Tet⁺ CD8 T cells.

Single Qa-1–FL9-PE⁺ Qa-1–FL9-APC⁺ cells were sorted and subjected to sequencing for TCRα and TCRβ. TCRα and TCRβ pairs were then analyzed based on their TCR V gene segments. Alignment of TCRα (**A**) and TCRβ (**B**) sequences obtained from Qa-1–FL9 Tet⁺ single cells. TCR affinity to Qa-1–FL9 complex and TCR Vα and Vβ gene usage by each Qa-1–FL9 Tet⁺ CD8 T cell are shown on the right. TRAV9N3 (Vα3.2) and/or TRBV12.1/2 (Vβ5.1/2) expressing CD8 T cell clones are highlighted.

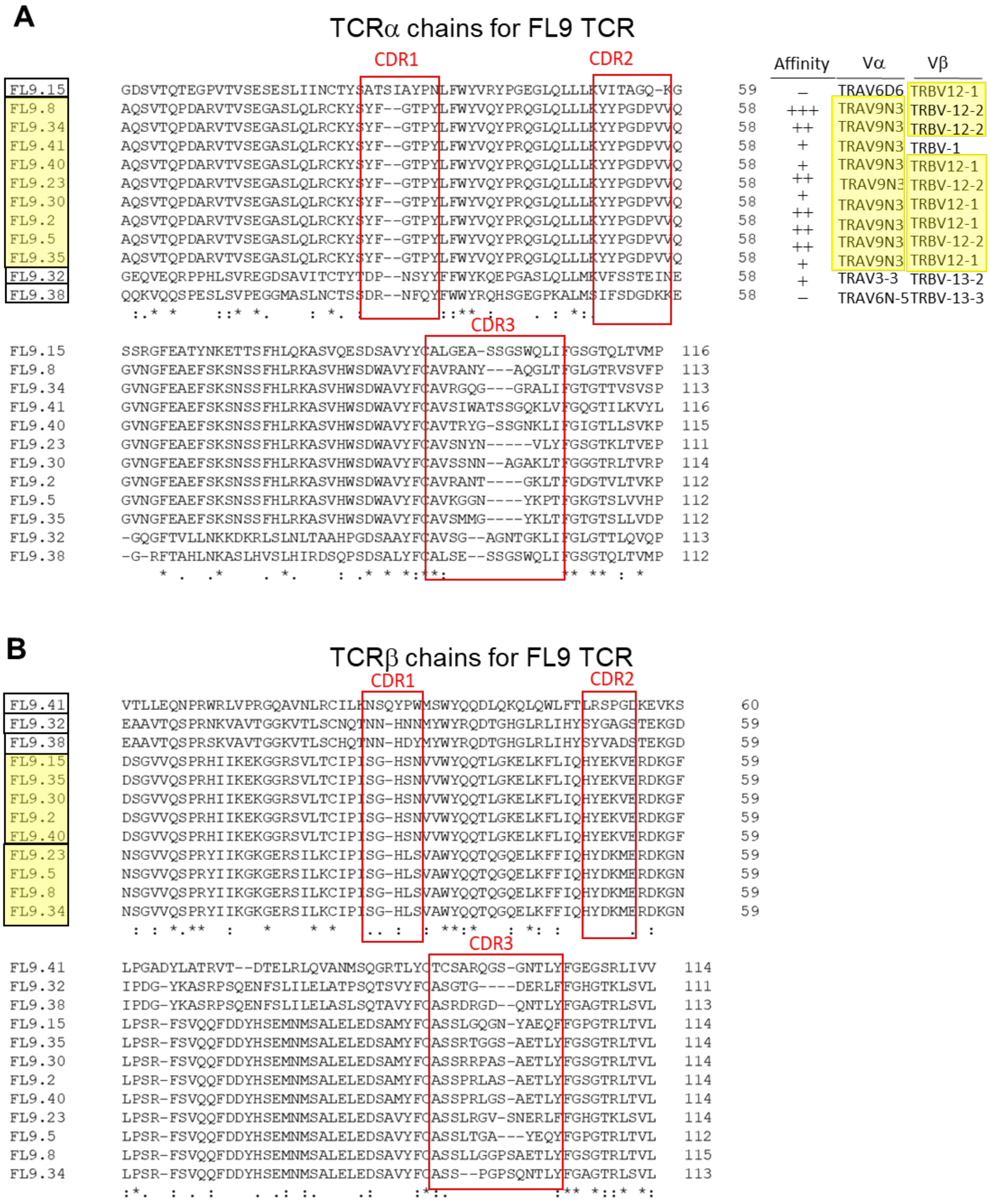

Supplemental Figure 2. TCR repertoire of Qa-1–Hsp60 Tet⁺ CD8 T cells.

Single Qa-1–Hsp60-PE⁺Qa-1–Hsp60-APC⁺ cells were sorted and subjected to sequencing for TCRα and TCR TCRα and TCRβ pairs were analyzed based on their TCR V gene segments. Alignment of TCRα (**A**) and TCRβ (**B**) sequences obtained from Qa-1–Hsp60 Tet⁺ single cells. TCR Vα and Vβ gene usage by each Qa-1–Hsp60 Tet⁺ CD8 T cell are shown on the right. TRAV9N3 (Vα3.2) and/or TRBV12.1/2 (Vβ5.1/2) expressing CD8 T cell clones are highlighted.

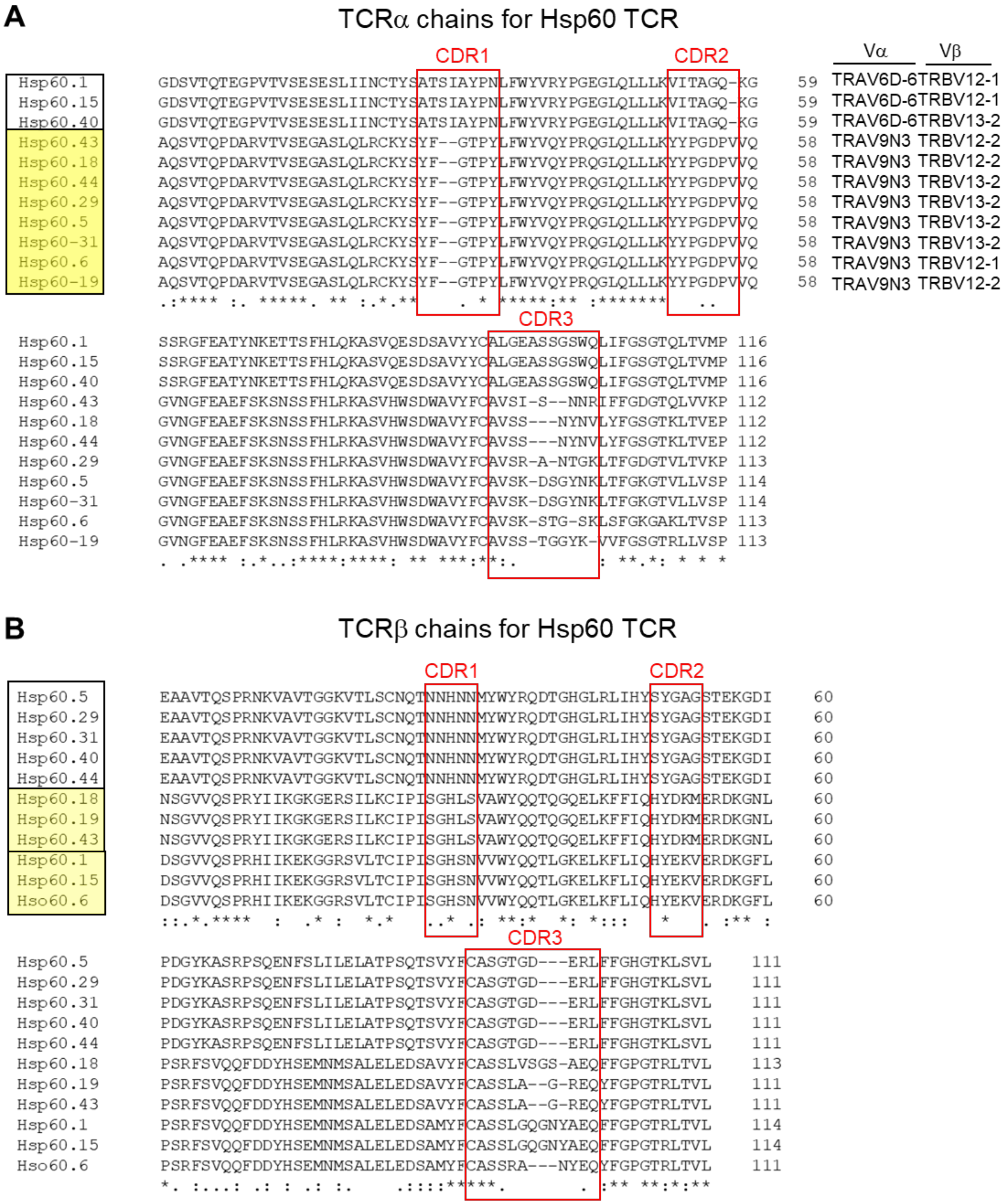

Supplementary Figure 3. Control study of TCR chain usage:

A single-cell sorting technique was utilized to collect two types of T cells: $TCR\beta^+CD8^+CD44^+CD122^+Ly49^+$ T cells (45 cells) and $TCR\beta^+CD8^+CD44^+CD122^+Ly49^-$ T cells (45 cells). The sorted cells underwent RT-PCR amplification of TCR α and β chains, followed by electrophoresis. Paired amplification was observed in 37 out of 45 $Ly49^+$ cells (82%) and 33 out of 45 $Ly49^-$ cells (73.3%). Sanger sequencing analysis revealed the specific TCR chain usage. Among the $Ly49^+$ cells, 4 cells exhibited TRAV9N-3 (Vα3.2) gene presence (10.8%), while only 1 cell displayed TRAV9N-3 positivity (3%) in the $Ly49^-$ population.

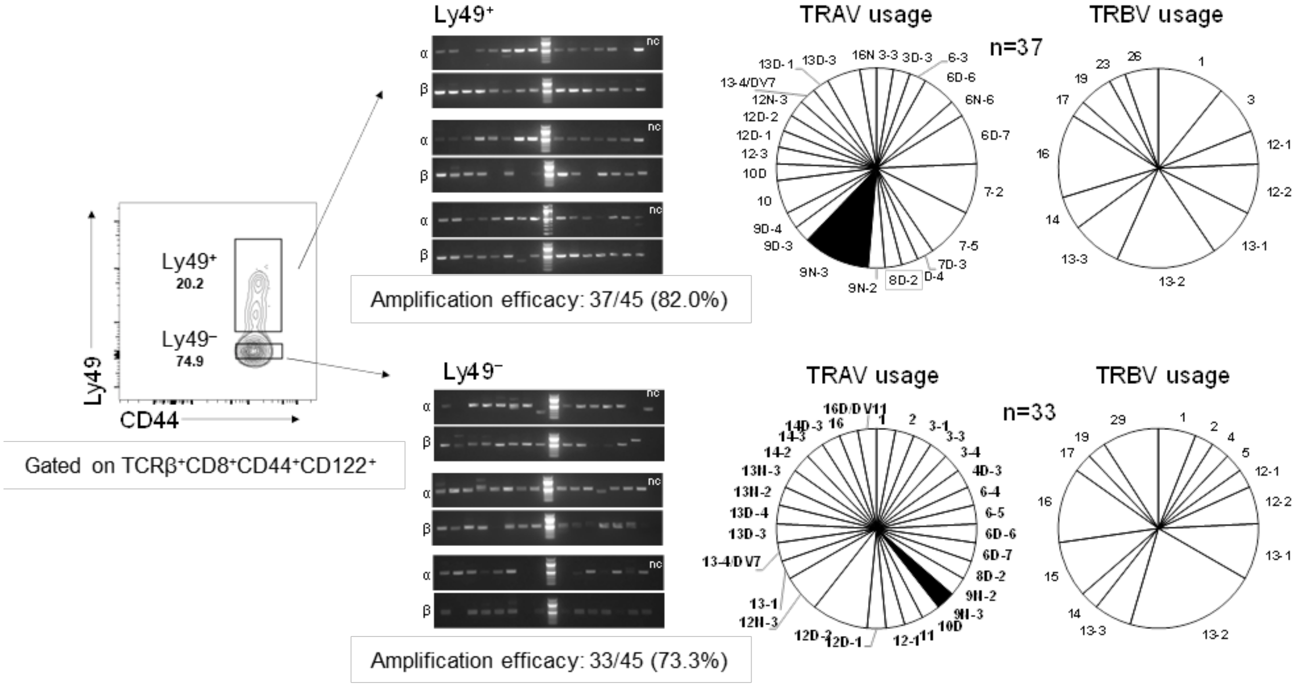

Supplemental Figure 4. Gating strategy for detection of Ly49^{+}Vα3.2^{+}Vβ5^{+} CD8 T cells.

A) Gating strategy for Vα3.2^{+}Vβ5^{+} CD8 cell detection. Frequency of Vα3.2^{+}Vβ5^{+} CD8 cells in the Ly49^{+} and Ly49^{-} CD8 cells. **B**) Frequency of Vα3.2^{+} or Vβ5^{+} cells within Ly49^{+} CD8 cells in spleen and LNs of WT B6, Qa-1.D227K KI (DK) and Qa-1 KO (KO) mice at 8 weeks of age.

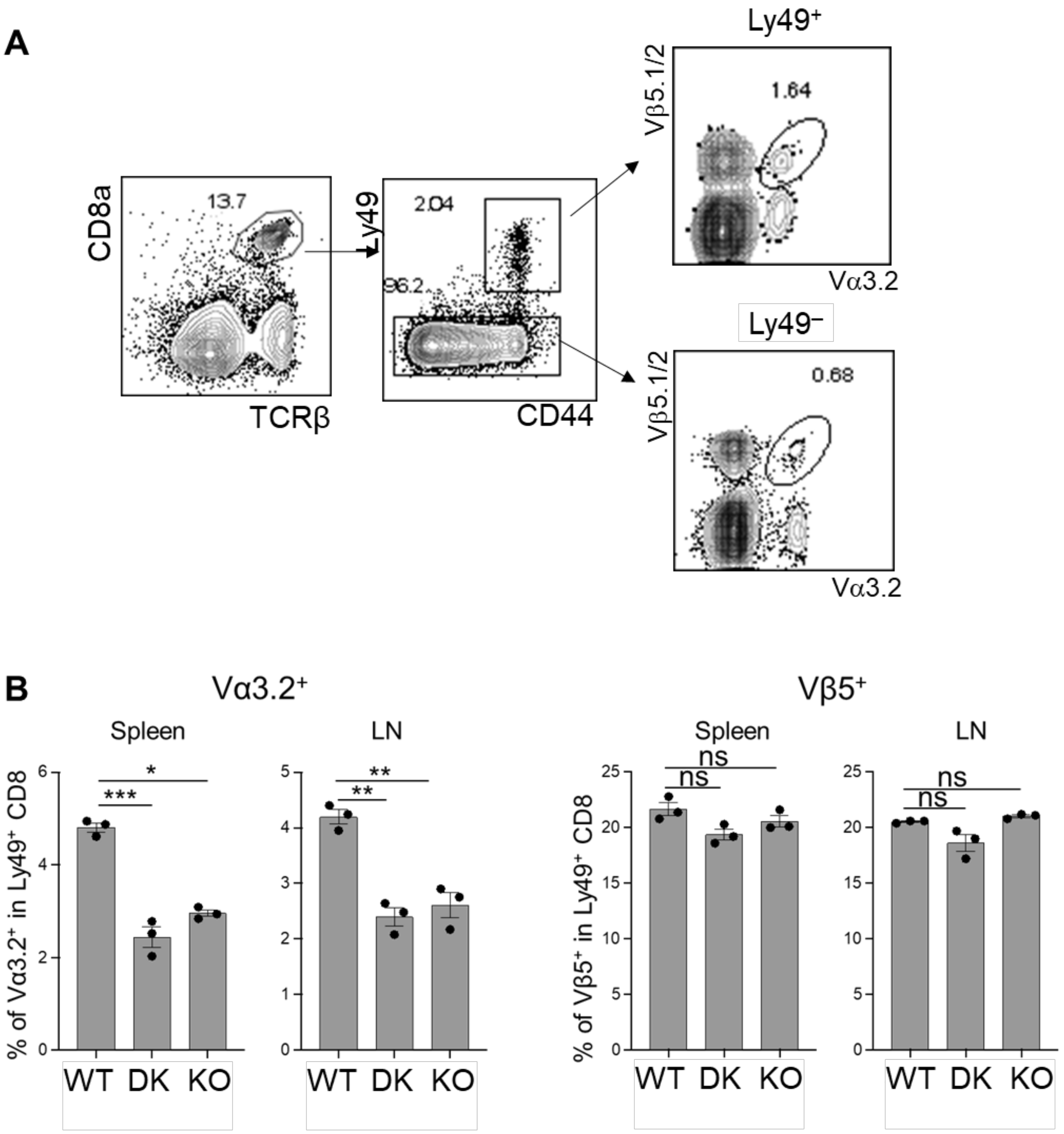

Supplemental Figure 5. Generation of FL9 TCR⁺ hybridoma and analysis of their responsiveness to FL9 peptide.

A) 58C$\alpha^{-}\beta^{-}$ hybridomas were transduced with OT-I TCR or FL9 TCRs that were identified from single cell TCR sequencing from Qa-1–FL9 tet⁺ CD8 T cells. Correct folding and assembly of transduced TCRs and their expected specificity were tested by staining with anti-CD3, anti-TCR Vβ and Qa-1–Hsp60 or Qa-1–FL9 tetramers. **B**) Qa-1–FL9 tetramer-based detection of TCRs on 58C hybridomas that were transduced with individual TCR pairs (sequences shown in Suppl. Fig. 1) isolated from Qa-1–FL9-specific single CD8 T cells. **C**) Responsiveness of FL9 TCR⁺ hybridoma to increasing concentrations of FL9 peptide depicted by surface CD69 expression.

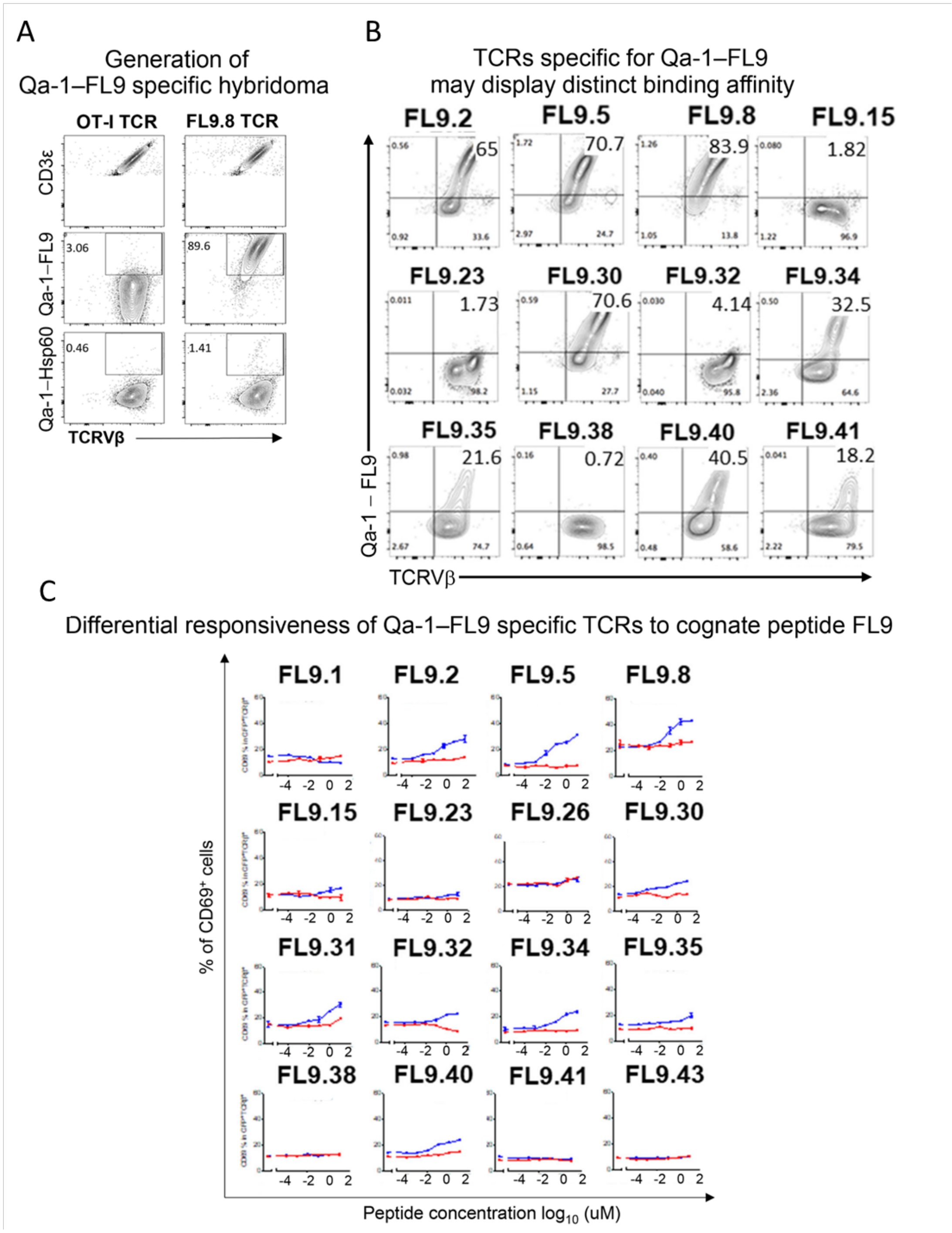

Supplemental Figure 6. **Qa-1-dependent differentiation of FL9 T cells.**

A) Tg TCR$^+$ cells (Vα3.2$^+$Vβ5$^+$) in TCR$^+$ thymocytes and percent of active-Caspase 3$^+$PD1$^+$ cells in DP (CD4$^+$CD8$^+$) thymocytes in OT-I → WT B6, FL9.8 Tg → WT B6 BM chimera 8 wks after BM reconstitution. **B**) Ki67 and CD44 expression by OT-I and FL9.8 TCR Tg CD8$^+$ T cells was measured as an indication of Ag encounter in spleen and liver of OT-I → WT B6 and FL9.8 Tg → WT B6 BM chimeras 8 wks after BM reconstitution. OT-1 is also used as a control in Fig. 1H. **C)** TCR and CD8 expression on polyclonal non-Tg CD8 cells, OT-I, FL9.2 and FL9.8 Tg T cells.

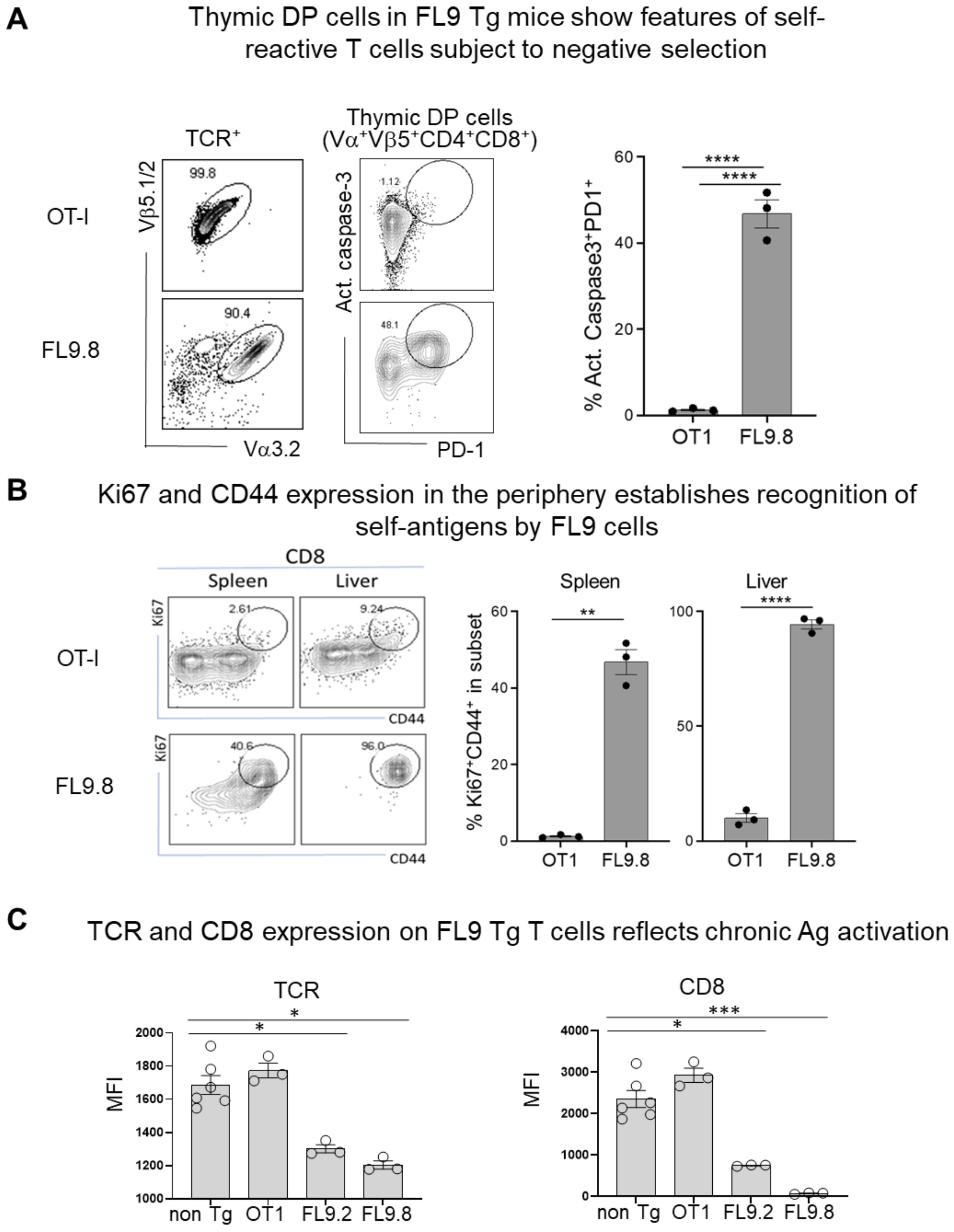

Supplemental Figure 7. NKG2D expression by FL9 TCR Tg T cells.

Acquisition of NKG2D expression by FL9.2 (**A**) and FL9.8 (**B**) T cells with age. Percentage of NKG2D$^+$ cells within CD8$\alpha\beta^+$ FL9 T cells from spleen, LN and liver in FL9 TCR Tg mice at the ages of 18 days, 9 wks and 4 mos.

A

Increased expression NKG2D with age by FL9.2 T cells

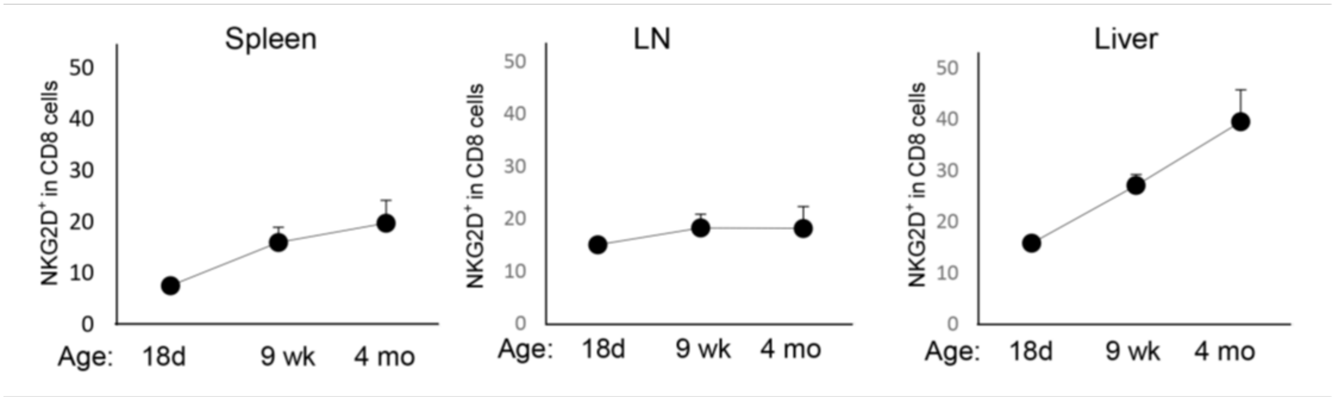

B

Increased expression of NKG2D with age by FL9.8 T cells

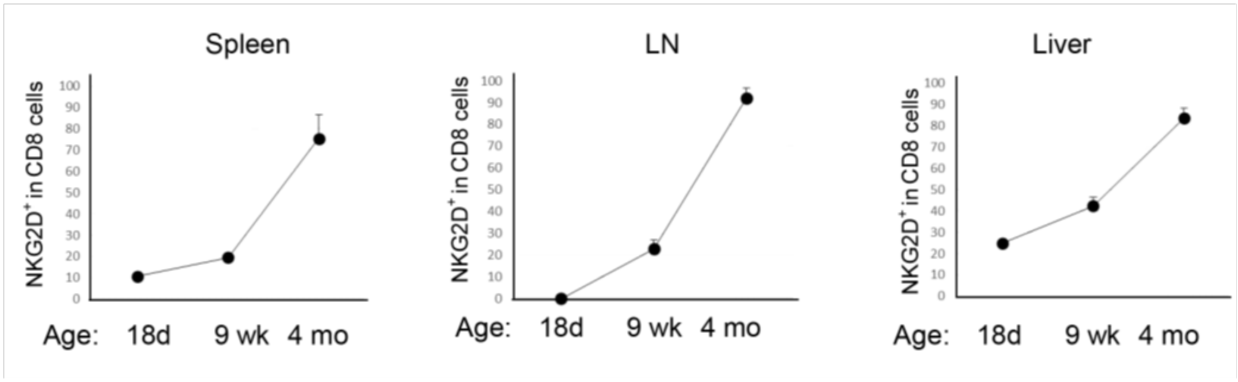

Supplemental Figure 8. Qa-1 dependent phenotype acquisition by FL9.8 Tg T cells.

A) Frequency of Vα3.2⁺Vβ5⁺ T cells in TCRβ⁺ cells from FL9.8 TCR Tg mice on Qa-1 WT and KO backgrounds. Representative FACS plots for detection of Vα3.2⁺Vβ5⁺ cells in spleen are shown on the left. **B**) Expression of CD44 and NKG2D by FL9.8 TCR Tg T cells in spleen and LNs of Qa-1 WT and Qa-1 KO mice. Representative FACS plots for NKG2D⁺CD44⁺ cells in spleens of FL9.8 TCR Tg mice are shown on the left. **C)** Expression of Ki67 by FL9.8 T cells in the LNs of WT.FL9.8 TCR Tg and Qa-1$^{-/-}$.FL9.8 TCR Tg mice at 8 wks of age.

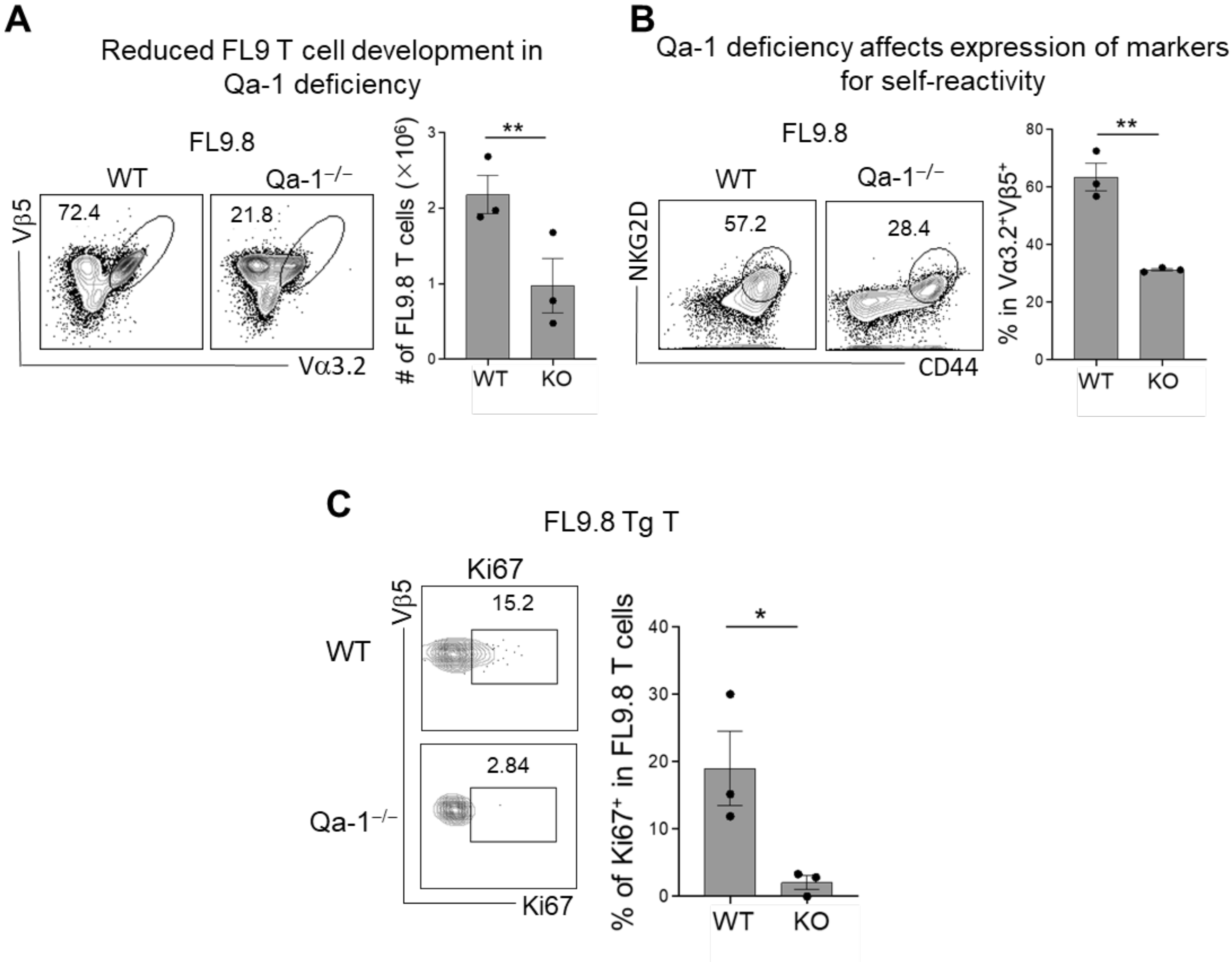

Supplemental Figure 9. FL9 Tg CD8 T cells recognize activated CD4 T cells.

A) ConA-stimulated CD4 cells from WT B6, Qa-1.D227K KI (DK), K^bD^b KO and ERAAP KO mice were co-cultured with FL9.8 T cells isolated from spleen and LNs of FL9.8 TCR Tg mice. After 20 hrs, CD69 expression on FL9 Tg T cells was measured as a readout of TCR stimulation. ConA-stimulated WT CD4 cells loaded with FL9 peptide were used as a positive control. **B)** OT-I or FL9.8 TCR^+ hybridomas were co-cultured with ConA-stimulated CD4 cells from WT or ERAAP KO mice**.** Stimulation of OT-1 or FL9 TCR was measured according to CD69 expression by hybridoma cells. Mean ± SEM is indicated. ****P < 0.0001, ***P < 0.001, **P < 0.01, *P < 0.05.

A

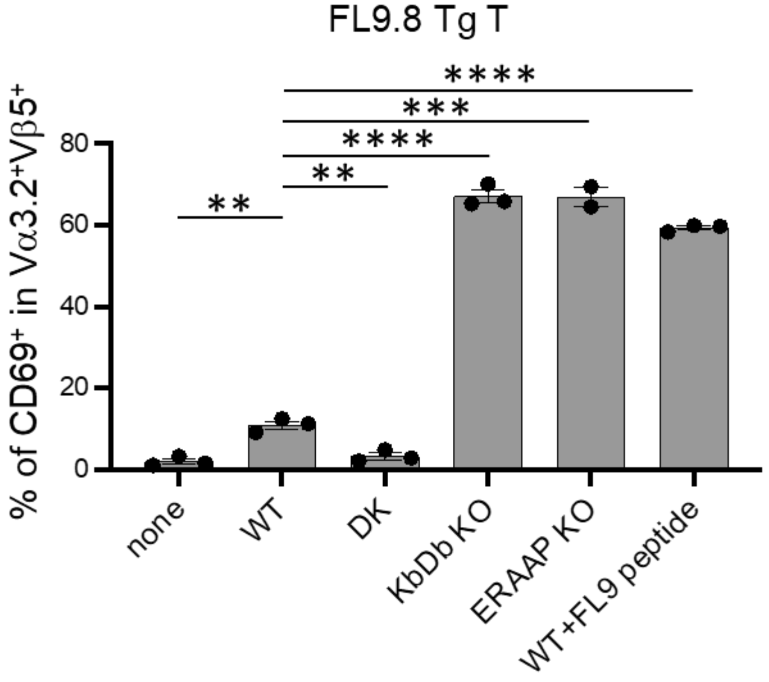

B

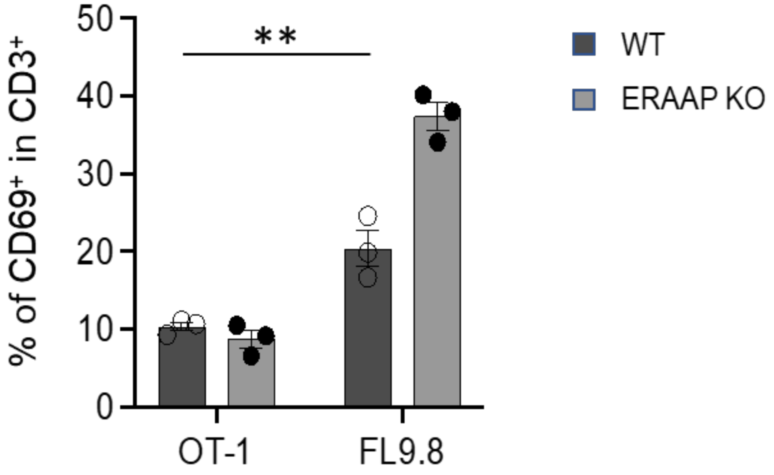

Supplemental Figure 10. Antibody-dependent depletion of Vα3.2$^+$ Ly49$^+$ CD8 T cells.

WT B6 and B6.Qa-1.D227K mice were immunized with Ova/CFA along with injection of rat IgG2b or anti-Vα3.2 Abs at day 0. These mice were boosted with Ova/IFA on day 8 and also injected with Abs. At day 15, the presence of Vα3.2$^+$Vβ5$^+$ cells was assessed in total T, CD8 T and Ly49$^+$ CD8 T cells. Shown are plots from analysis of spleen cells from WT mice.

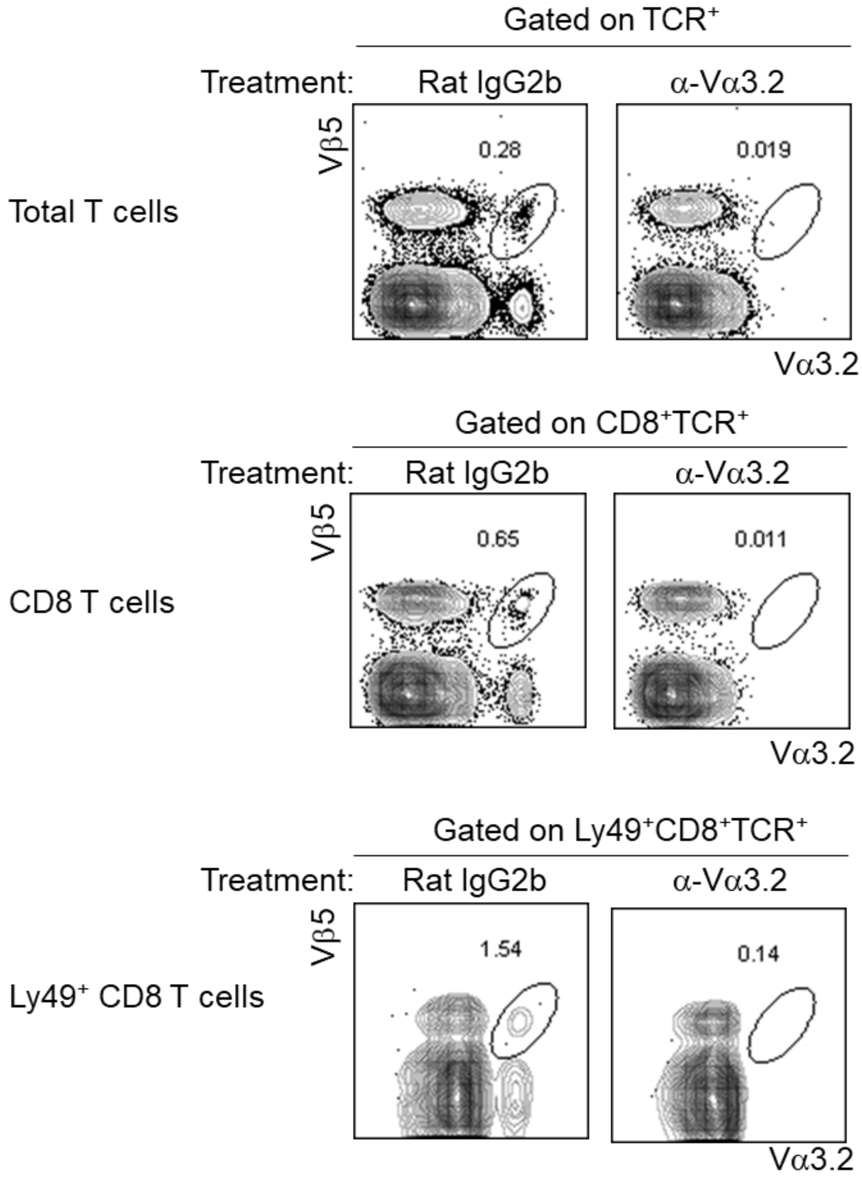

Supplemental Figure 11. Failure of native FL9 peptide to stimulate FL9.2 T cells.

CD45.1⁺ B6 hosts were adoptively transferred with FL9.2 T cells and immunized i.p. with FL9 in CFA or no peptide (CFA alone) on day 0. Three days later, activation (CD69) and proliferation (Ki67) of FL9.2 T cells were measured.

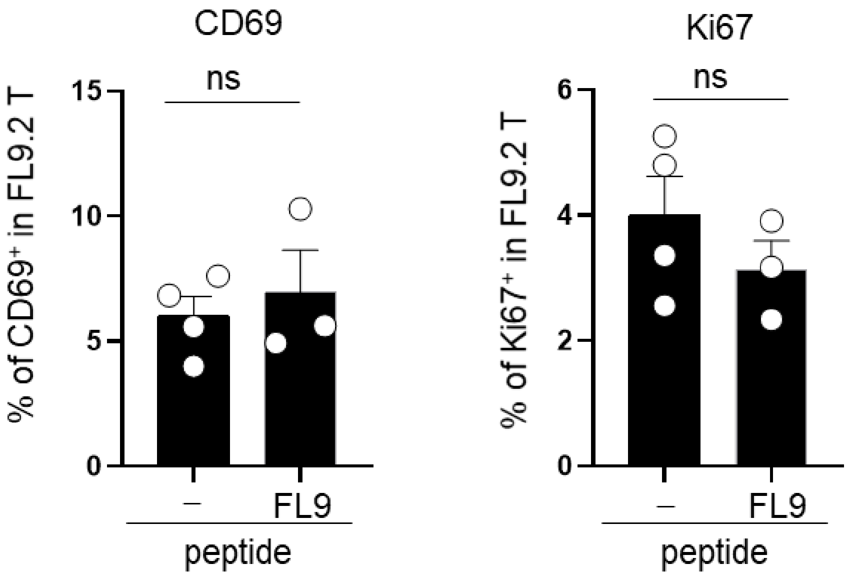

Supplemental Figure 12. Identification of superagonist peptides.

Activation of FL9.8 T cells after stimulation with FL9 variants selected from the library screen described in Fig. 4. Dose-dependent activation of FL9.8 T cells was measured by culturing FL9.8 T cells with EL4 (Qa-1⁺) at various concentrations of the indicated peptides (0, 1, 3 and 10μg/ml).

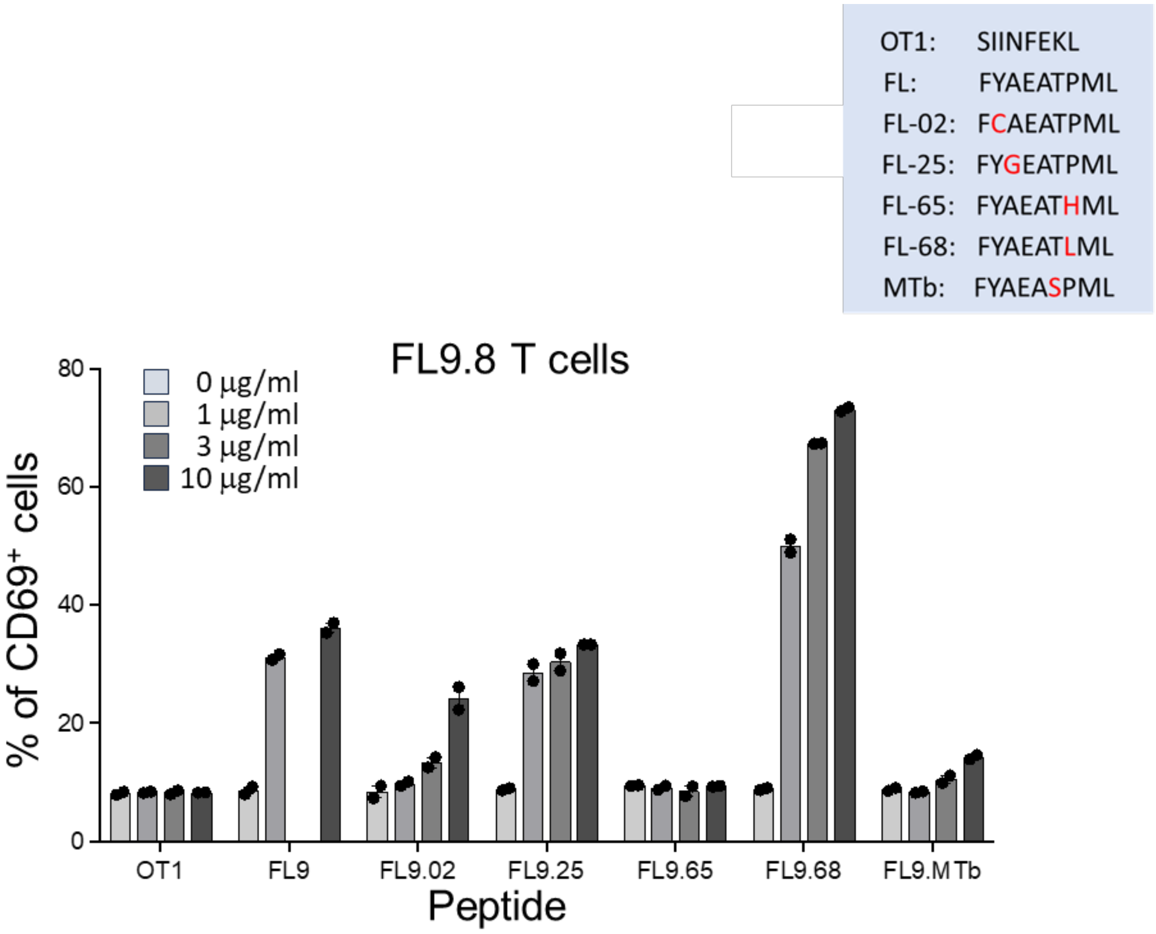

Supplemental Table 1. Primer sequences for the amplification of TCRα and TCRβ sequences

Primers for TCRα	
AL-1	AAGGATCCGAATTCCTGCAGGatgctgcagatgtgggggtttg
AL-10	AAGGATCCGAATTCCTGCAGGatgaagacatcccttcacac
AL-10-4	AAGGATCCGAATTCCTGCAGGatggataaaacatcccttc
AL-10D	AAGGATCCGAATTCCTGCAGGatggattaagacatcccttc
AL-11	AAGGATCCGAATTCCTGCAGGatgaaaaagYgcctKagtgcctg
AL-14-1	AAGGATCCGAATTCCTGCAGGatggacaMgaWcctgacag
AL-14D-1	AAGGATCCGAATTCCTGCAGGatggacaagattctgacag
AL-15-1	AAGGATCCGAATTCCTGCAGGatgcctcctcacagcctgYtc
AL-15-2	AAGGATCCGAATTCCTGCAGGatgcctcctcagagcctgctc
AL-16	AAGGATCCGAATTCCTGCAGGatgctgattctaagcctgYtg
AL-17	AAGGATCCGAATTCCTGCAGGatgttccYagtgaccattctg
AL-19	AAGGATCCGAATTCCTGCAGGatgactggcttcctgaag
AL-2	AAGGATCCGAATTCCTGCAGGatgaagcaggtggcaaaagt
AL-21	AAGGATCCGAATTCCTGCAGGatgggatgtgtgagtggaattg
AL-3-1	AAGGATCCGAATTCCTGCAGGatgaagacRgtgactgga
AL-3-4	AAGGATCCGAATTCCTGCAGGatgaaaacagtgRctgga
AL-4-2	AAGGATCCGAATTCCTGCAGGatggagaggagcccgggaac
AL-4-3	AAGGATCCGAATTCCTGCAGGatgSagaggaacctggttg
AL-4-4	AAGGATCCGAATTCCTGCAGGatgcagaggaacctgggag
AL-5-1	AAGGATCCGAATTCCTGCAGGatgaagacagctattcatg
AL-5D-4	AAGGATCCGAATTCCTGCAGGatgaaaacataYgctcctac
AL-6-1	AAGGATCCGAATTCCTGCAGGatgaacWattcYccagcttta
AL-6-4	AAGGATCCGAATTCCTGCAGGatgaaYacttctccagYttta
AL-6-5	AAGGATCCGAATTCCTGCAGGatgaacctttRtcctgaactg
AL-6-6	AAGGATCCGAATTCCTGCAGGatgRactcttctccaggcttc
AL-7-1	AAGGATCCGAATTCCTGCAGGatgaagtccttgtgtgtttc
AL-7-2	AAGGATCCGAATTCCTGCAGGatgaaatccttKagtRtttc
AL-7-6	AAGGATCCGAATTCCTGCAGGatgcattccttacatgtttc
AL-7D-4	AAGGATCCGAATTCCTGCAGGatggtacaaacacagatgttc
AL-7D-4-	AAGGATCCGAATTCCTGCAGGatgaaatccttgagtgtttta
AL-8-1	AAGGATCCGAATTCCTGCAGGatgcacagcctcctRgggttg
AL-8-1-3	AAGGATCCGAATTCCTGCAGGatgcacagcctcctggggttg
AL-8-2	AAGGATCCGAATTCCTGCAGGatgaacagattcctgggaata
AL-9-1	AAGGATCCGAATTCCTGCAGGatgctcctggtYctcatctc
AL-9-2	AAGGATCCGAATTCCTGCAGGatgctcctggYgctcctcYcag
AL-9D-4	AAGGATCCGAATTCCTGCAGGatgctcctggcactcctcccag
AL-12-1	AAGGATCCGAATTCCTGCAGGatgcgtcctgDcacctgctc
AL-12D-1	AAGGATCCGAATTCCTGCAGGatgcgtcctgtcacctcctc
AL-13-1	AAGGATCCGAATTCCTGCAGGatgaacaggctgctgtgctctc
AL-13-2	AAGGATCCGAATTCCTGCAGGatgaagaggctgctgtgttctc
AL-13D-1	AAGGATCCGAATTCCTGCAGGatgaagaggctgctgagctctc

Primers for TCRβ	
BL-1	AAGGATCCGAATTCCTGCAGGatgtggcagttttgcattctg
BL-2	AAGGATCCGAATTCCTGCAGGatgggctccattttcctcag
BL-3	AAGGATCCGAATTCCTGCAGGatggatatctggcttctag
BL-4	AAGGATCCGAATTCCTGCAGGatgggctgtaggctcctaag
BL-5	AAGGATCCGAATTCCTGCAGGatgagctgcaggcttctc
BL-12-1	AAGGATCCGAATTCCTGCAGGatgtctaacactgtcctcgctg
BL-12-2	AAGGATCCGAATTCCTGCAGGatgtctaacactgccttccctg
BL-13-1	AAGGATCCGAATTCCTGCAGGatgggctccaggctctttc
BL-13-2	AAGGATCCGAATTCCTGCAGGatgggctccaggctcttcttc
BL-13-3	AAGGATCCGAATTCCTGCAGGatgggctccagactcttctttg
BL-14	AAGGATCCGAATTCCTGCAGGatgggcaccaggcttcttg
BL-15	AAGGATCCGAATTCCTGCAGGatgggcatccagaccctctg
BL-16	AAGGATCCGAATTCCTGCAGGatggcccccaggctcctttttctg
BL-17	AAGGATCCGAATTCCTGCAGGatggatcctagacttctttg
BL-19	AAGGATCCGAATTCCTGCAGGatgaacaagtgggttttctg
BL-20	AAGGATCCGAATTCCTGCAGGatgttactgcttctattacttc
BL-23	AAGGATCCGAATTCCTGCAGGatgggtgcacggctcatttg
BL-24	AAGGATCCGAATTCCTGCAGGatgggtgcaagactgctctg
BL-26	AAGGATCCGAATTCCTGCAGGatggctacaaggctcctctg
BL-29	AAGGATCCGAATTCCTGCAGGatgagagttaggctcatctctg
BL-30	AAGGATCCGAATTCCTGCAGGatgtggacattcctgctacttc
BL-31	AAGGATCCGAATTCCTGCAGGatgctgtactctctccttgc
